# Light-entrained chromatin priming poises rapid metamorphosis in a marine sponge

**DOI:** 10.7554/eLife.111060

**Published:** 2026-10-27

**Authors:** Huifang Yuan, Oceane Blard, Zac Pujic, Bernard M Degnan, Sandie M Degnan

**Affiliations:** 1 Centre for Marine Science and School of the Environment, The University of Queensland Brisbane Australia; Heidelberg University Germany; Max Planck Institute for Biology Germany

**Keywords:** *Amphimedon*, metamorphosis, gene expression, gene regulation, larva, transcription factor, Other

## Abstract

The most widespread animal life cycle includes a planktonic larval stage that is environmentally induced to settle and rapidly metamorphose into a benthic juvenile. Although this transition is critical for survival, how planetary cues such as light interact with local ecological signals to prepare the genome for such rapid reprogramming remains poorly understood. Here, using the sponge *Amphimedon queenslandica*, we integrate time-resolved transcriptomic and chromatin accessibility profiling across larval competence, settlement, and the first hours of metamorphosis. We show that diminishing light at sunset is associated with extensive chromatin remodelling in swimming larvae prior to settlement despite relatively modest changes in gene expression. Upon environmental induction by a coralline alga, metamorphosis is accompanied by the rapid and transient activation of a large suite of deeply conserved transcription factors (TFs), including AP-1/bZIP family members, whose binding motifs are enriched in newly accessible regulatory regions near differentially expressed genes. Larvae prevented from experiencing sunset by constant light fail to settle and instead adopt an alternative transcriptional and chromatin state characterised by widespread loss of accessibility and repression of competence-associated TFs, including the circadian regulator CLOCK and other bHLH-PAS factors. These observations support a model in which light-entrained transcriptional activity establishes an anticipatory, permissive chromatin landscape that enables immediate and coordinated transcriptional reprogramming upon environmental induction. We propose that such chromatin priming may represent a broadly deployed regulatory strategy underlying the speed and robustness of metamorphosis in biphasic animal life cycles.

## Introduction

The biphasic pelagobenthic life cycle is widespread in early-diverging animal lineages (sponges and cnidarians) and in molluscs, echinoderms, annelids and several other bilaterian phyla, suggesting it evolved before metazoan cladogenesis (Degnan and Degnan, 2010; Degnan and Degnan, 2006; Hadfield, 2000; Hedgpeth and Jagersten, 1974; Martín-Zamora et al., 2023; Nielsen, 1998; Rieger, 1994). This ancient aquatic life cycle begins with endogenously regulated embryogenesis that produces a ciliated planktonic larva, and culminates with settlement and metamorphosis into a benthic juvenile. In contrast to embryogenesis, metamorphosis is characterised by three defining features: environmental induction, coordinated transformation of one complex body plan into another, and a dramatic ecological transition (Hadfield, 2000; Hedgpeth and Jagersten, 1974; Nielsen, 1998). Despite the scale of morphological and physiological change required, in diverse marine invertebrates this transition occurs strikingly quickly as a means to enhance post-settlement survival (Conaco et al., 2012; Hadfield, 2000; Heyland and Moroz, 2006; Wray, 1999).

Marine larvae are strongly influenced by planetary constants, including daily, tidal, lunar and seasonal rhythms, with gametes or larvae being released at a species-specific time. Most larvae also have developmentally regulated behaviours that enable settlement to be induced by species-specific biochemical cues that are associated with a favourable settlement site for adult survival and reproduction (Bishop et al., 2006a; Ettinger-Epstein et al., 2008; Hadfield et al., 2001; Hodin et al., 2015; Randall et al., 2024). The capacity to respond to these cues – widely called ‘larval competence’ – develops while larvae swim in the plankton over timescales ranging from minutes to weeks, depending on the species (Bishop et al., 2006b; Hadfield, 2000; Hodin et al., 2015; Jackson et al., 2002; Randall et al., 2024; Say and Degnan, 2020). Together, these traits indicate that larvae across disparate animal phyla have evolved to integrate constant planetary signals with local ecological cues to optimise dispersal, settlement success and subsequent survival.

Despite the prevalence and ecological importance of this life cycle, it remains unclear how the metazoan genome integrates planetary and ecological signals to regulate the acquisition of larval competence, the decision to settle, and the rapid remodelling of the body plan at the onset of metamorphosis. Here, we address this problem by exploiting the experimental tractability of the sponge *Amphimedon queenslandica*, in which larval release, competence, settlement, and metamorphosis are well characterised and readily manipulated (Blard et al., 2026; Conaco et al., 2012; Degnan et al., 2015; Degnan and Degnan, 2010; Jindrich et al., 2017; Nakanishi et al., 2015; Say and Degnan, 2020; Sogabe et al., 2016; Ueda et al., 2016; Wong et al., 2022). We experimentally induce competent larvae to settle on a naturally co-occurring coralline alga under natural and perturbed light conditions and profile chromatin accessibility and gene expression in the same individuals during the first hours of metamorphosis. This approach allows us to reveal that larvae anticipate impending settlement through diurnal light cues and the deployment of pioneer-like transcription factors (TFs) that establish a permissive chromatin landscape prior to environmental induction. Given that sponges diverged from other animal lineages more than 700 million years ago, elucidating these mechanisms provides insight into how deeply conserved gene regulatory programmes enabled the evolution of rapid, environmentally induced metamorphosis in the ancestral biphasic animal life cycle.

## Results

### Dramatic changes in gene expression in the first hour of metamorphosis

**Figure 1.**
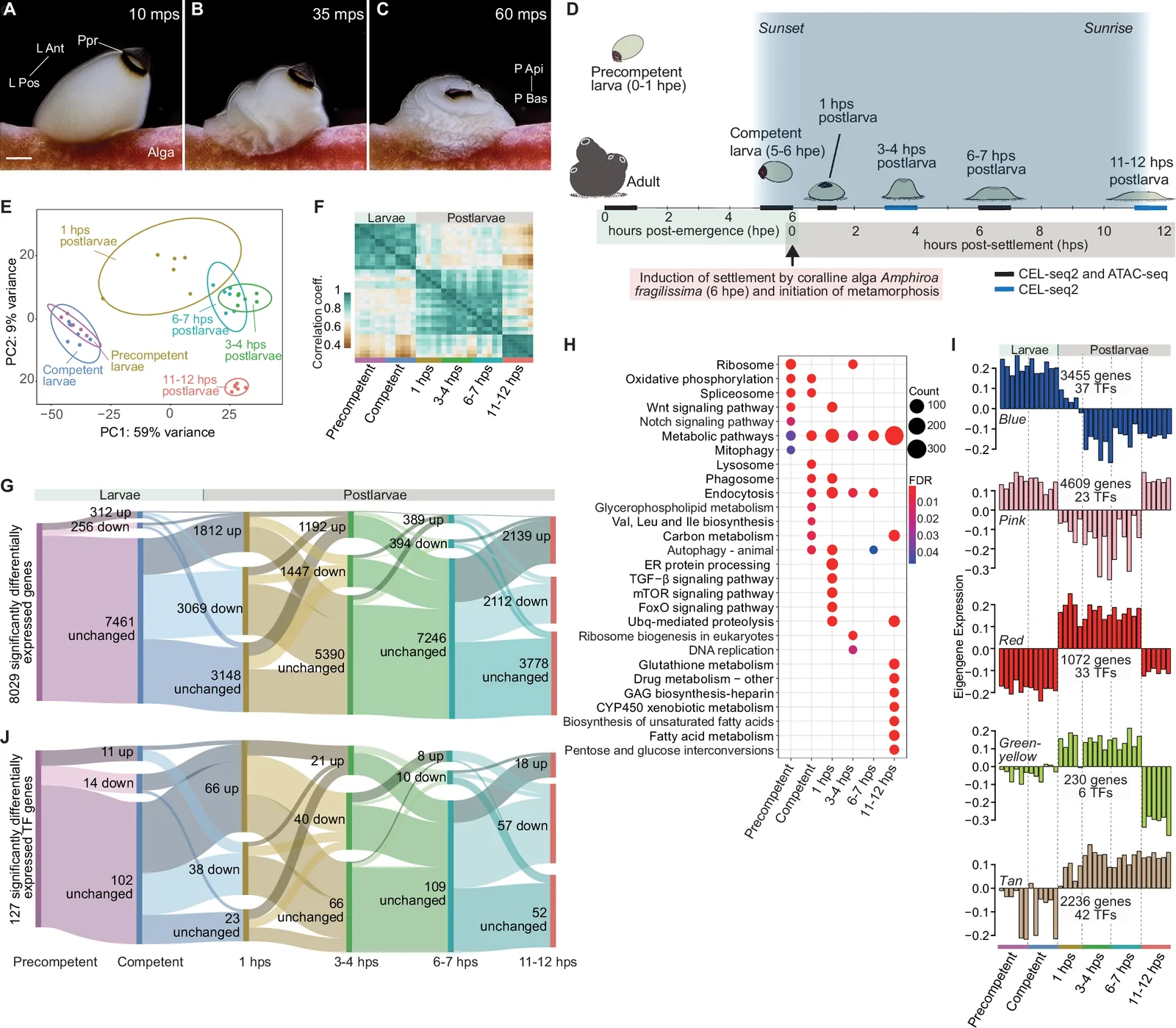
Gene expression during larval development and the initiation of sponge metamorphosis. (**A–C**) Photomicrographs of the first hour of metamorphosis after competent *A. queenslandica* larvae settle on the coralline alga *A. fragilissima* (Alga). L Ant and L Pos, larval anterior–posterior axis; P Api and P Bas, postlarval apical–basal axis; Ppr, posterior pigment ring; mps, minutes post-settlement; scale bar, 100 µm (see Figure 1—video 1; Blard et al., 2026). (**D**) Timeline of larval and early postlarval developmental stages analysed using CEL-seq2 and ATAC-seq (see Methods). Larvae become competent to respond to an inductive cue associated with *A. fragilissima* just after sunset, 5–6 hr after emerging from the adult sponge (Degnan and Degnan, 2010; Say and Degnan, 2020). (**E**) Principal component analysis (PCA) of CEL-seq2 transcriptomes with 95% confidence level ellipses shown; *n* = 6 for each stage. (**F**) Hierarchical clustered heatmap of Pearson correlation coefficients of replicated larval and postlarval transcriptomes based on DESeq2-normalised counts of the 8029 significantly differentially expressed genes (DESeq2; p-adj <0.1). (**G**) Alluvial plot showing dynamics of differentially expressed genes through larval development and early metamorphosis. (**H**) Top 10 significantly enriched KEGG pathways (FDR <0.05) based on significantly upregulated genes at each developmental stage; GO-MWU analysis of upregulated genes reveal stage-specific enrichments largely consistent with the KEGG analysis (Figure 1—figure supplement 1; Supplementary file 4). FDR, false discovery rate. (**I**) Weighted gene co-expression network analysis (WGCNA) co-expression modules that comprise genes that are down- (blue, pink) and up- (red, yellow-green, tan) regulated at the start of metamorphosis. Number of coding genes and transcription factors (TFs) are shown. (**J**) Alluvial plot showing dynamics of 127 significantly differentially expressed TF genes as per **G**.

*A. queenslandica* larvae emerge from adult sponges in the early afternoon and become competent to settle on the coralline alga *Amphiroa fragilissima* 5–6 hr later, shortly after sunset (Degnan and Degnan, 2010; Say and Degnan, 2020). We have shown previously that diminishing light at sunset is necessary for larvae to respond to the algal cue under normal conditions and to initiate settlement and metamorphosis (Say and Degnan, 2020). The first hour following settlement is characterised by extensive cellular reprogramming and the rapid dissolution of the larval swimming anteroposterior axis (Figure 1A–C; Figure 1—video 1; Blard et al., 2026; Degnan et al., 2015; Leys and Degnan, 2002; Nakanishi et al., 2015; Nakanishi et al., 2014; Sogabe et al., 2016).

**Figure 1—figure supplement 1.**
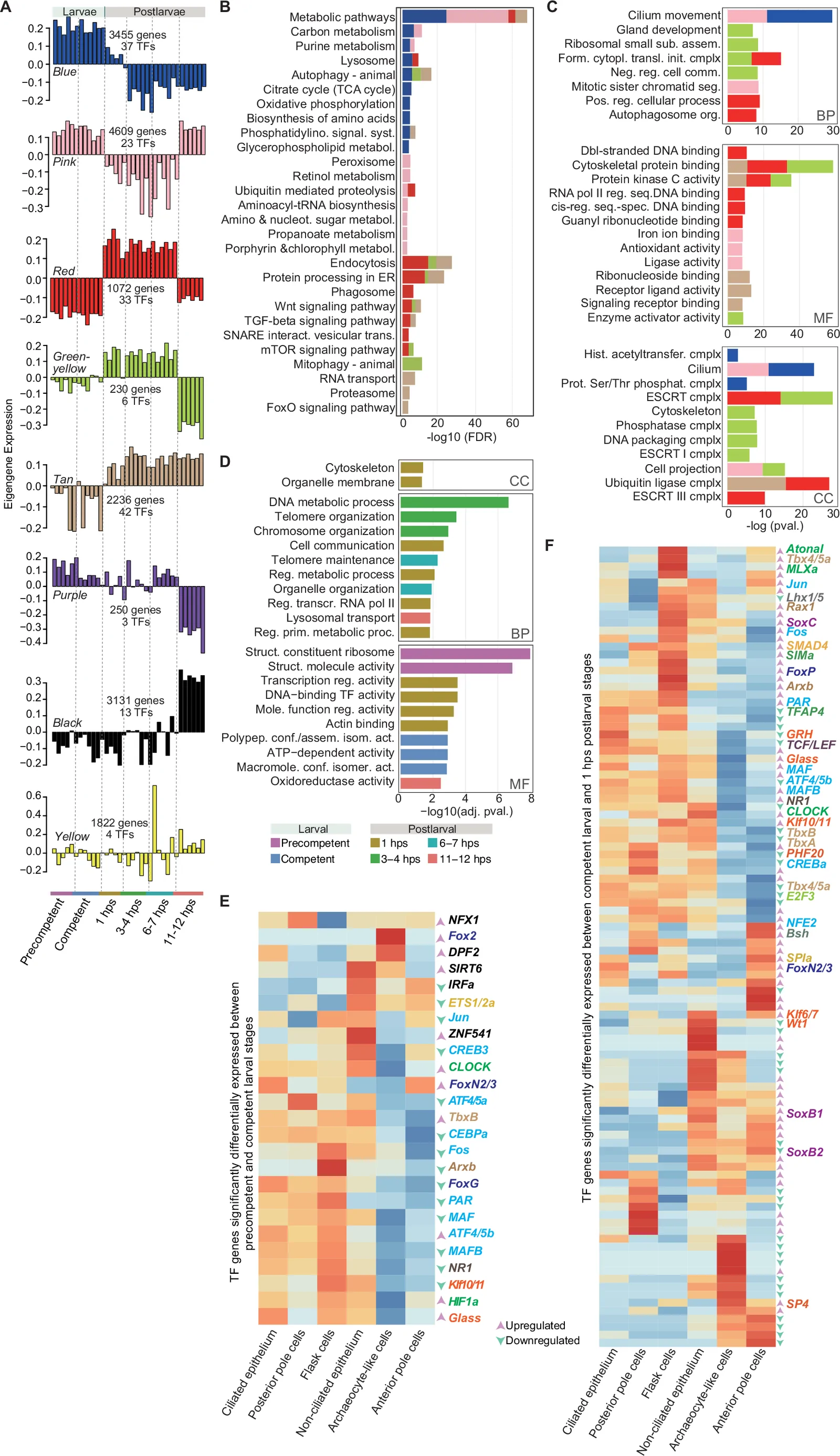
Larval and postlarval gene expression. (**A**) Weighted gene co-expression network analysis (WGCNA) co-expression modules of genes expressed through larval and postlarval development. Number of coding genes and transcription factors (TFs) are shown. KEGG (**B**) and GO-MWU (**C**) analysis of WGCNA co-expression modules that comprise genes that are down- (blue, pink) and up- (red, yellow-green, tan) regulated at the start of metamorphosis. BP, biological process; CC, cellular component; and MF, molecular function. (**D**) Developmentally enriched GO-MWU terms (−log p-value; maximum of 10 shown) based on significantly upregulated genes at each developmental stage. (**E, F**) Scaled heatmaps of expression of TFs in larval cell types previously defined using scRNA-seq (Sebé-Pedrós et al., 2018). (**E**) TF genes significantly differentially expressed between precompetent and competent larval stages. (**F**) TF genes significantly differentially expressed between competent larval and 1 hps postlarval stages. Highly expressed TFs are in bold, and arrows in E and F show whether the TF is up- or downregulated between successive stages.

**Figure 1—video 1.** Time-lapse video of the first 1–2 hr of metamorphosis, beginning within 10 min of an *A. queenslandica* larva attaching to a fragment of the coralline alga *A. fragilissima*. Images were obtained every 3 min for approximately 2 hr with each time point consisting of a z-stack of 14 images at an interval of 25 µm. The movie is 180x normal speed.

To investigate transcriptional and chromatin changes associated with the acquisition of competence and the onset of metamorphosis at sunset, we analysed transcriptomes (six biological replicates per stage) from precompetent larvae, competent larvae, and postlarvae at 1, 3–4, 6–7, and 11–12 hours post-settlement (hps) (see Methods; Figure 1D; Supplementary file 1). Across this time course, 8029 genes were differentially expressed (DESeq2; p-adj <0.1). Swimming larvae, early postlarvae (1, 3–4, and 6–7 hps) and later postlarvae (11–12 hps) showed transcriptionally distinct states (Figure 1E–G; Supplementary files 2 and 3).

The most pronounced transcriptional shift occurred within the first hour of metamorphosis, with 4881 genes differentially expressed from competent larval to 1 hps postlarval stages; of these, 63% were downregulated (Figure 1G; Supplementary file 3). These repressed genes were enriched for diverse metabolic processes (Figure 1H; Supplementary file 4). In contrast, genes transiently upregulated during this interval were involved in Wnt, TGF-β, mTOR, and FoxO signalling, and endocytosis (Figure 1H; Supplementary file 4). Together, these results indicate a rapid shift from a larval physiological state to a metamorphic developmental programme, coincident with the onset of widespread reprogramming of larval cell types and reorganisation of the body plan (Blard et al., 2026). By 11–12 hps, gene expression shifted back towards metabolic processes, reflecting the establishment of a new postlarval physiological state (Figure 1H; Supplementary file 4).

Weighted gene co-expression network analysis (WGCNA) assigned 69% of expressed genes to five co-expression modules, typified by either up- or downregulation between competent larval and 1 hps postlarval stages (Figure 1I; Figure 1—figure supplement 1; Supplementary file 5). These modules reinforce the conclusion drawn from the DESeq2 analysis that the first hour after settlement represents the most transcriptionally dynamic phase, marked by coordinated repression of larval metabolic programmes and activation of developmental signalling pathways (Figure 1—figure supplement 1).

### Conserved TFs are expressed at exceptionally high levels during metamorphosis

**Figure 2.**
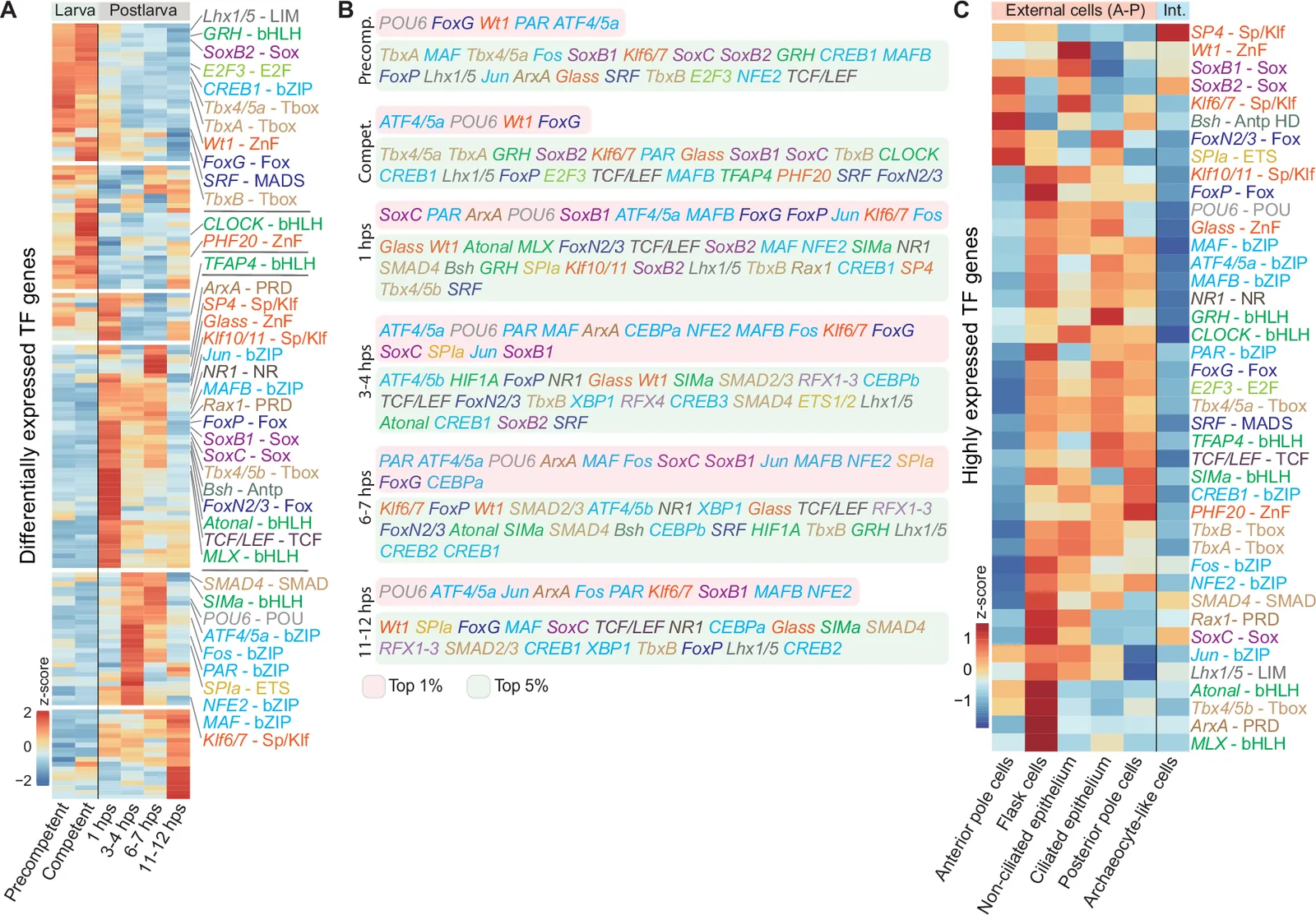
Dynamic and high-level expression of transcription factor (TF) genes during larval development and early metamorphosis. (**A**) Scaled heatmap of 159 TF genes expressed during larval and postlarval development, of which 127 are significantly differentially expressed between at least two successive stages. The 40 most highly expressed TFs, all of which are among the top 5% of the most-highly expressed genes, are annotated to the right of the heatmap. TF gene names are followed by their gene family name, which is colour-coded into families; this colour-coding is used in all figures. (**B**) Lists of TF genes in the order of their level of expression for all larval and postlarval stages. TFs in the pink and green boxes are among the top 1 and 5% of all expressed genes, respectively (Supplementary file 6). (**C**) Scaled heatmap of the 40 most highly expressed TFs in larval cell types (Sebé-Pedrós et al., 2018). External epithelial cells are to the left – cell types ordered left-to-right based on their enrichment level along the anterior–posterior (A–P) axis; internal archaeocyte-like cells are to the right (Supplementary file 7).

There are 127 TFs that are differentially expressed during larval development and early metamorphosis, 52 of which rank in the top 5% of expressed genes (Figures 1J and 2A, B; Supplementary files 3 and 6). These highly expressed TFs span multiple conserved families, including bZIP, bHLH, Tbox, Fox, Sp/Klf, homeobox, and Sox. All but one of these highly expressed TFs (the exception being the constitutively expressed SRF) share three features: (1) they are differentially expressed either during the acquisition of larval competence or within the first hour of metamorphosis; (2) they belong to WGCNA modules that change sharply at the onset of metamorphosis; and (3) they are significantly downregulated by 11–12 hps (Supplementary files 5 and 6). This coordinated temporal pattern is consistent with a transient but potent regulatory phase commencing at settlement and associated with large-scale reprogramming of larval cell states in the first hours of metamorphosis.

**Figure 2—figure supplement 1.**
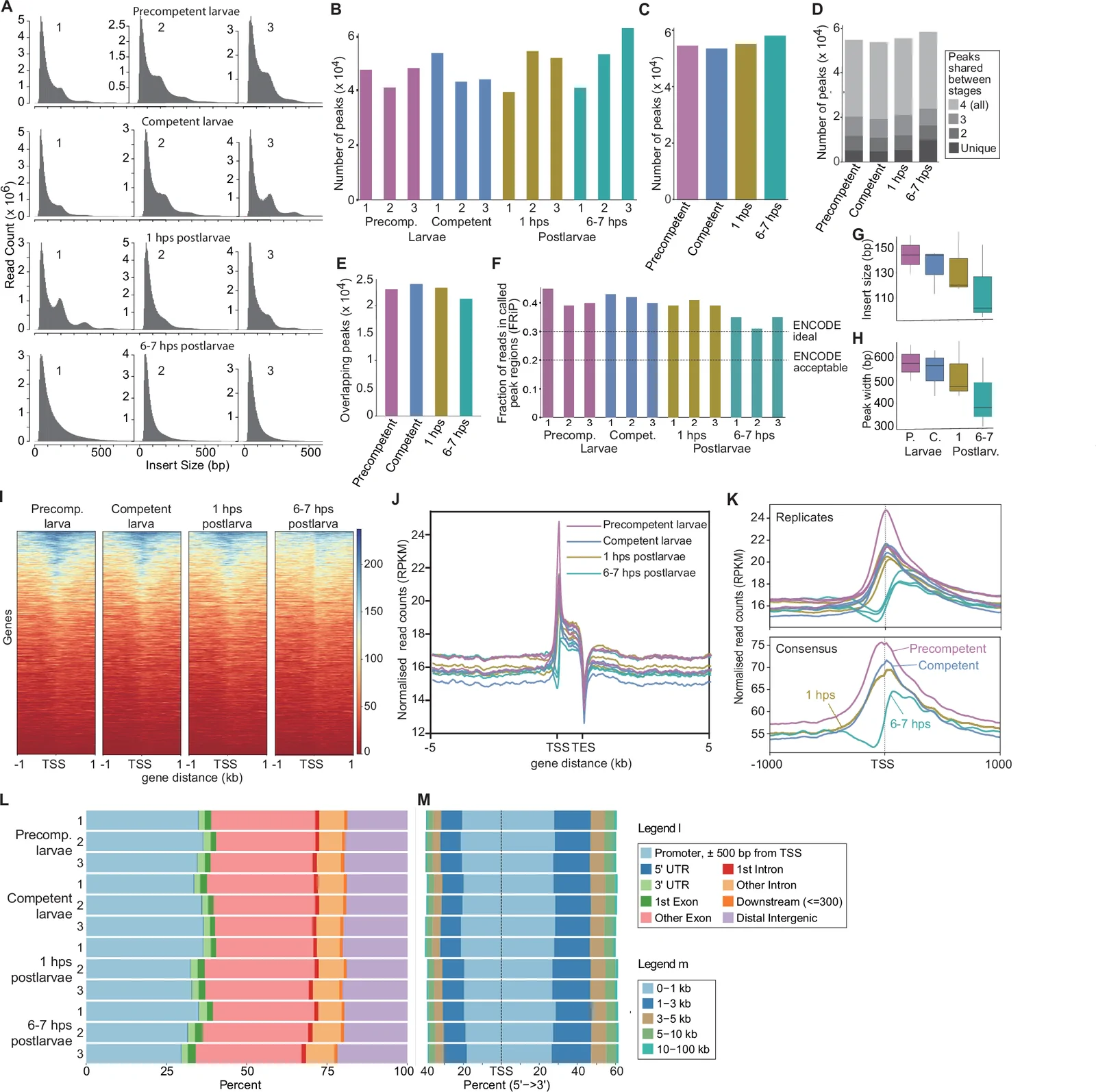
ATAC-seq analysis of *A. queenslandica* larval and early postlarval chromatin. Analyses were performed on cells dissociated from three individual precompetent and competent larvae, and 1 and 6–7 hps postlarvae. (**A**) Insert size distribution of ATAC-seq libraries. Three biological replicates for each stage shown. (**B**) Bar graph of number of ATAC-seq peaks in each replicate. Developmental stages are colour-coded as per Figure 1. (**C**) Bar graph of the total number of open chromatin regions (OCRs) identified per developmental stage. (**D**) Bar graph of the number of ATAC-seq peaks shared between larval and postlarval stages. Most peaks are present in all four stages. (**E**) Bar graph of the number of ATAC-seq peaks shared between stages. (**F**) ATAC-seq FRiP scores for all replicates. Dotted lines, ENCODE standards (https://www.encodeproject.org/atac-seq/). (**G**) Boxplot of ATAC-seq peak insert size, calculated using the arithmetic mean across replicates for each stage. (**H**) Boxplot of ATAC-seq peak width size, calculated as per H. (**I**) Heatmaps of total ATAC-seq reads for each developmental stage within 1 kb up- and downstream of TSSs of all genes. (**J**) Normalised ATAC-seq read density from TSS to transcription end site (TES) and 5 kb up- and downstream for all genes in each replicate and stage, showing enrichment and depletion at TSSs and TESs, respectively. (**K**) Chromatin accessibility read density 1 kb up- and downstream of the TSS, by stage. Top panel plots the read density for three replicate ATAC-seq analyses for each larval and postlarval stage. Bottom panel shows the consensus plot for each stage. (**L**) The percentage of ATAC-seq peaks mapping to genomic features for larval and postlarval replicates. Promoter region is defined as a region within 500 bp of the TSS. Downstream is defined as ≤300 bp of the end of a gene. (**M**) The percentage of ATAC-seq peaks up- and downstream of the TSS for larval and postlarval replicates, partitioned into classes based on distance from the TSS.

As larvae acquire competence to settle at sunset, 11 TFs are upregulated and 14 are downregulated (Figures 1J and 2A, B; Supplementary file 6). Among the upregulated and highly expressed TFs are two bHLH factors (*CLOCK* and *HIF-1*), the bZIP *ATF4/5*, two Fox TFs (*FoxN2/3* and sponge-specific *Fox 2*), *TboxB*, and the zinc-finger (ZnF) TF *Glass. CLOCK* exhibits the most pronounced transient expression at competence, increasing relative to precompetent larvae (p-adj = 5.2e−6) and decreasing at 1 hps (p-adj = 1.5e−15) (Figure 2A; Supplementary file 6). In parallel, a sponge nuclear receptor (*NR1*) and multiple bZIP factors are significantly downregulated, including the highly expressed AP-1 components *Fos* and *Jun*, and several of their potential partners (*CEBP*, *CREB*, *PAR*, *MAF*, and *NFE2*) (Jindrich et al., 2017; Reinke et al., 2013; Rodríguez-Martínez et al., 2017). These TFs, together with most other highly expressed TFs, are enriched in externally facing larval cell types – ciliated and non-ciliated epithelia and flask cells – that are implicated in detecting the algal inductive cue and that undergo rapid morphological and functional transformations during the first hour of metamorphosis (Figure 2C; Figure 1—figure supplement 1; and Supplementary file 7; Blard et al., 2026; Leys and Degnan, 2002; Nakanishi et al., 2014; Nakanishi et al., 2015; Sebé-Pedrós et al., 2018; Sogabe et al., 2016; Ueda et al., 2016).

The sharp transcriptional reorganisation that characterises the first hour of metamorphosis is accompanied by a striking and selective induction of TFs. Of the 66 TFs significantly upregulated in the first hour of metamorphosis, 48 rank within the top 5% of all expressed genes (Figure 2A, B; Supplementary file 6). Notably, AP-1 and the bZIP factors that are downregulated at competence are rapidly and strongly induced (Figure 2A; Supplementary file 6). In addition, TFs with deeply conserved roles in animal development (multiple Sox, bHLH, Fox, SMAD, Tbox and homeobox members, and NR1) and innate immunity (NF-κB, STAT, and IRF) are transiently upregulated at the onset of metamorphosis (Figure 2A, B; Supplementary file 6). The rapid induction and exceptionally high expression of these TFs are consistent with a central role in coordinating environmentally induced cell state transitions and establishing a robust regulatory environment capable of driving the rapid transformation of the larval body plan into that of the filter-feeding juvenile.

Similar to the dynamics observed for developmental signalling pathways, nearly all of these TFs are transiently upregulated during the first hour of metamorphosis and subsequently downregulated between 6–7 and 11–12 hps (Figure 2A, B; Supplementary file 6). Together, the scale, synchrony and tempo of TF activation and repression are consistent with the genome of competent larvae being poised to respond immediately to the algal inductive cue, enabling a rapid and coordinated transcriptional response that underpins the extensive cell reprogramming that transforms the larval body plan.

### Open chromatin regions are enriched in binding motifs of highly expressed TFs

To determine how the *A. queenslandica* genome responds to the inductive algal cue, we profiled chromatin accessibility in precompetent and competent larvae, and 1 and 6–7 hps postlarvae (three biological replicates per stage) using an assay for transposase-accessible chromatin with high-throughput sequencing (ATAC-seq) (see Methods; Figure 1D; Buenrostro et al., 2015). We identified 60,716 non-overlapping open chromatin regions (OCRs; ATAC-seq peaks) that were highly reproducible across biological replicates and that overlapped with previously defined larval promoters and enhancers identified by ChIP-seq (Gaiti et al., 2017; Figure 2—figure supplement 1; Supplementary files 8 and 9). Consistent with patterns observed in *A. queenslandica* embryos and adults, as well as other animals (Cornejo-Páramo et al., 2022; Wong et al., 2020), approximately 80% of OCRs are located within protein-coding gene bodies or within 1 kb of transcription start or end sites (Figure 2—figure supplement 1).

**Figure 3.**
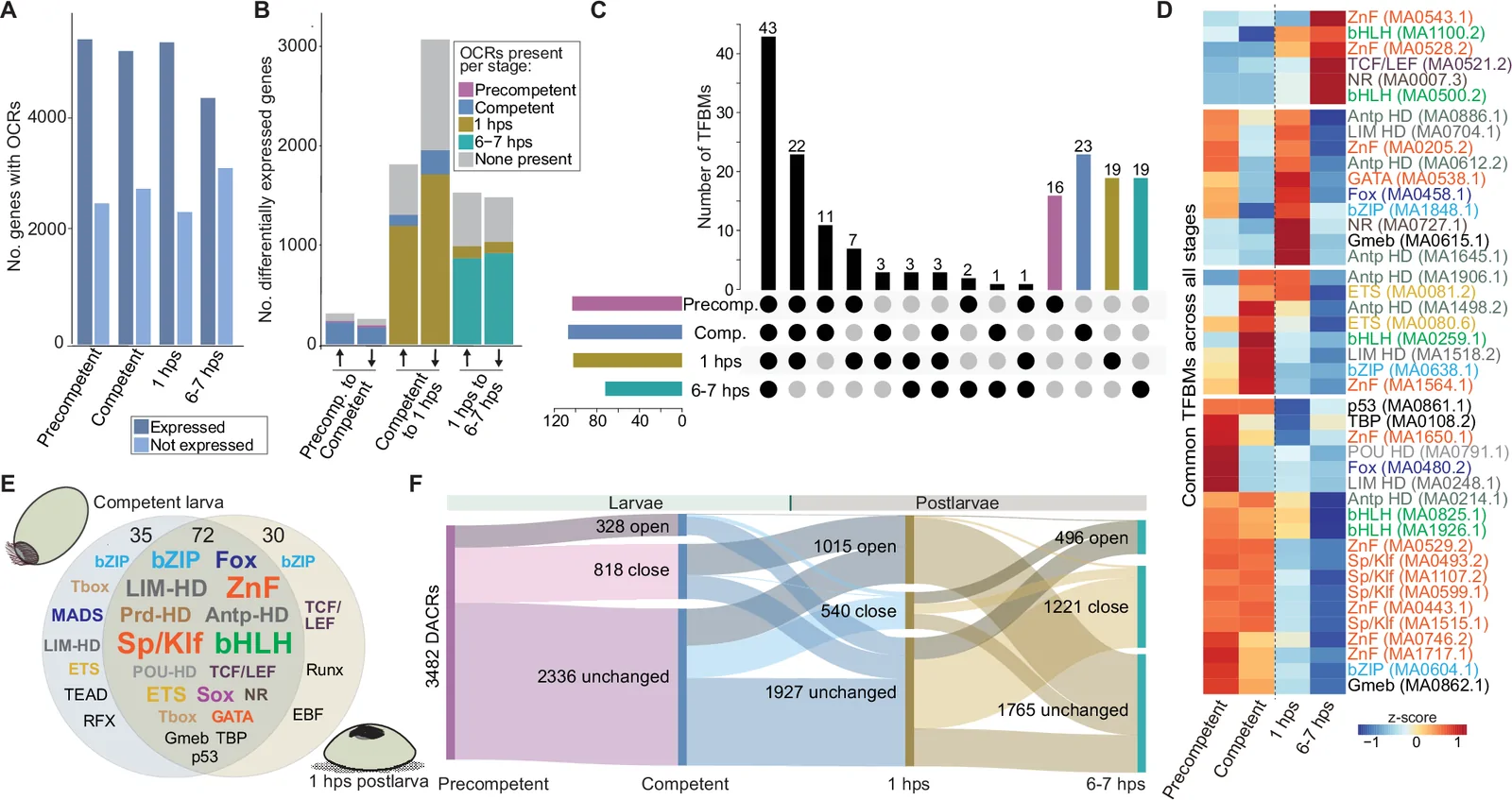
Open chromatin regions (OCRs) associated with expressed genes are enriched for binding sites of highly expressed transcription factors (TFs). (**A**) The number of OCRs associated with protein-coding genes expressed in larval and postlarval stages compared to genes not expressed at these stages. On average, there are 16,469 and 26,177 genes expressed and not expressed at these four stages, respectively (Supplementary file 10). (**B**) The number of significantly up- (↑) and down- (↓) regulated genes that have nearby OCRs. Colour coding within the bars show at which stage the OCRs first appear. (**C**) Upset plot showing the distribution of the 173 TFBMs shared and unique to each larval and postlarval stage. (**D**) Heatmap of the 43 TFBMs that are significantly enriched in all larval and postlarval stages. TFBMs that potentially interact with highly expressed TFs are colour-coded by TF class as per Figure 2A; black, TFBMs of other expressed TFs. JASPAR code follows TF class/family name to the right. (**E**) Venn diagram of the differential enhancement of TFBMs in OCRs associated with genes that are differentially expressed between competent larvae and 1 hps postlarvae, with the TFBM class and family name size scaled to prevalence (Supplementary file 12). (**F**) Alluvial plot showing differentially accessible chromatin regions (DACRs) across larval development and early metamorphosis. Open and close, chromatin accessibility significantly increases and decreases, respectively.

Between 67% and 86% of differentially expressed protein-coding genes and genes belonging to the five WGCNA co-expression modules have proximal OCRs, depending on the stage or module (Figure 3A, B; Figure 3—figure supplement 1; Supplementary files 10 and 11). There are approximately three times as many expressed genes that have OCRs compared to genes that are not expressed (Figure 3A). Expressed genes without nearby OCRs may be regulated by more distal *cis*-regulatory elements or, given the high gene density of the *A. queenslandica* genome (Fernandez-Valverde and Degnan, 2016), by OCRs associated with neighbouring genes.

**Figure 3—figure supplement 1.**
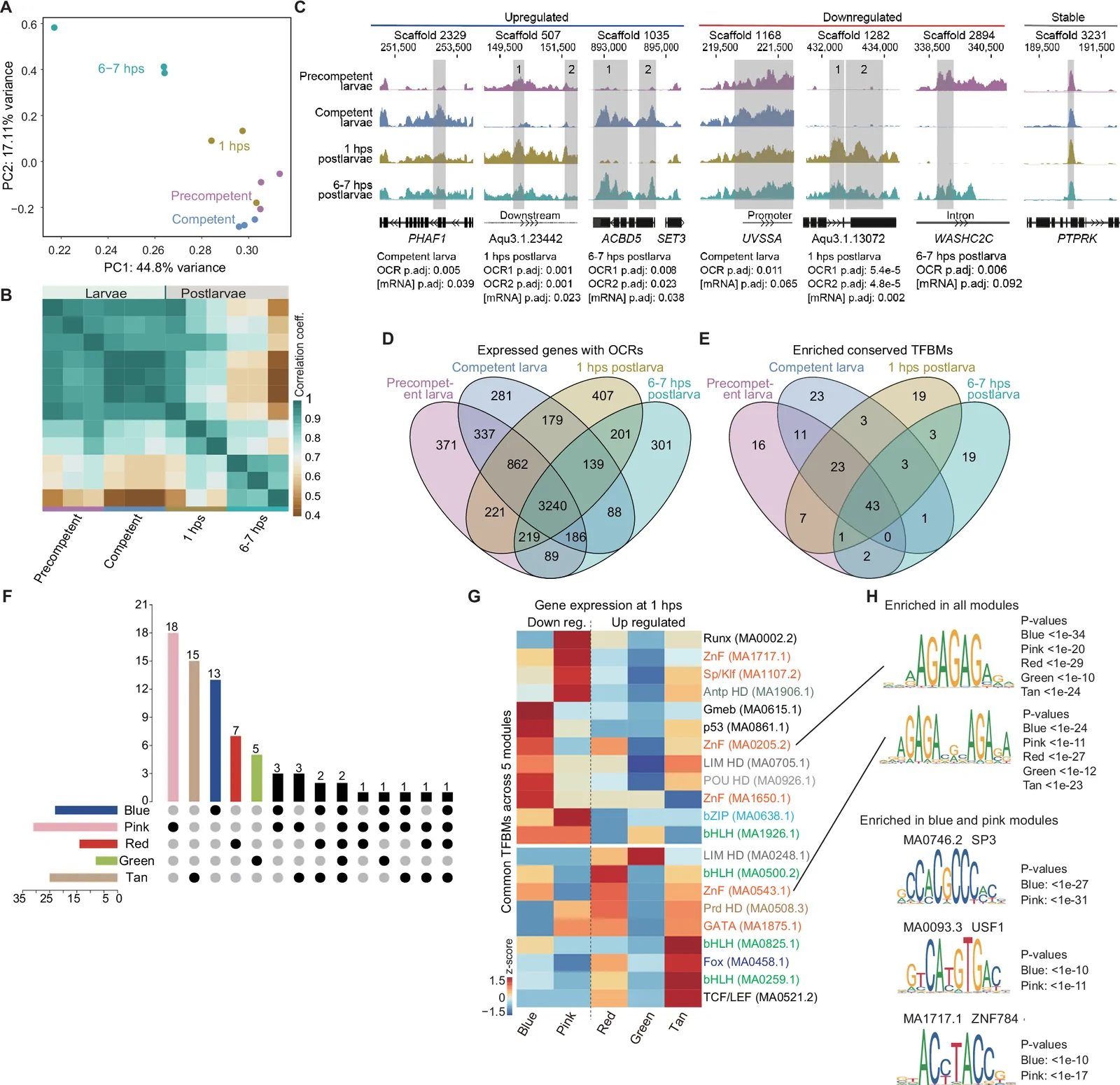
Chromatin dynamics through larval and early postlarval development. (**A**) PCA of the 3482 differentially accessible chromatin regions (DACRs) that significantly change in at least one larval or postlarval stage; *n* = 3 for all stages. (**B**) Hierarchical clustered heatmap of Pearson correlation of replicated larval and postlarval stages based on log_10_-transformed read counts of the 3482 DACRs. (**C**) Examples of the location and frequency of ATAC-seq reads mapped to the genome are shown and the regions of significant change are highlighted in grey. Scaffold locations of open chromatin regions (OCRs) are shown above and gene names below. Direction of transcription is depicted by chevrons and exons are depicted by black blocks. Below gene name is stage of development in which the gene is up- or downregulated, and the p-adjusted significance values of the OCRs and expression levels. *PHAF1*, *Phagosome Assembly Factor 1*; *ACBD5*, *Acyl-CoA binding domain-containing 5*; *UVSSA*, *UV stimulated scaffold protein A*; *WASHC2C*, *WASH complex subunit 2C*; *PTPRK*, *Protein tyrosine phosphatase receptor type K*. (**D**) Venn diagram of the OCRs unique to and shared between precompetent and competent larvae, and 1 and 6–7 hps postlarvae (Supplementary file 10). (**E**) Venn diagram of the conserved TFBMs unique to and shared between precompetent and competent larvae, and 1 and 6–7 hps postlarvae as determined by Homer (Supplementary file 10). (**F–H**) Analysis of TFBMs present in DACRs associated with the five weighted gene co-expression network analysis (WGCNA) modules whose genes change across the initiation of metamorphosis (Figure 1I and Supplementary file 11); module colours shown. (**F**) Upset plot of TFBMs unique to and shared between WGCNA modules. (**G**) Heatmap of the most common TFBMs shared across all modules. TFBMs that potentially interact with highly expressed transcription factors (TFs) is colour-coded by TF class as per Figure 2A; black, TFBMs of other expressed TFs. JASPAR code follows TF class/family name to the right. (**H**) Top, the two TFBMs that are significantly enriched in OCRs of all five WGCNA modules, with Homer p-values to the right. Bottom, three TFBMs enriched in OCRs associated with genes comprising blue and pink modules.

Previous studies have shown that sponge TFs can interact with bilaterian *cis*-regulatory elements and that sponge enhancers can drive developmental gene expression in vertebrates (Elliott et al., 2025; Richards et al., 2008; Wong et al., 2020). However, to avoid bilaterian-specific annotation biases – given that sponges and bilaterians diverged more than 700 million years ago – we restricted motif annotation to TF classes and families rather than individual TFs (see Methods). This analysis identified 173 TF-binding motifs (TFBMs) corresponding to TF classes and families that are significantly enriched in OCRs associated with genes expressed during larval development and early metamorphosis (Homer p-value <1e^–10^; Figure 3C; Supplementary file 10). Of these motifs, 78% correspond to predicted targets of the highly expressed TFs identified above, with an additional 15.6% attributable to highly expressed non-Sp/Klf ZnF TFs (93.6% total) (Figures 2A, B, 3D, E). These include 35 variants of the bHLH E-box motif (CACGTG), 40 homeodomain motifs (including POU, LIM and Antp), 17 Fox, 14 Sp/Klf, and 9 bZIP motifs. The enrichment of these 173 TFBMs varies dynamically across developmental stages and co-expression modules, consistent with stage-specific changes in chromatin accessibility during larval development and metamorphosis (Figure 3D–F; Figure 3—figure supplement 1; Supplementary files 10 and 11).

Across larval development and early metamorphosis, 3482 chromatin regions exhibit significant changes in accessibility (differentially accessible chromatin regions; DACRs). More than 56% of differentially expressed genes (DEGs) have OCRs at the same developmental stage in which they significantly change expression, while 4–8% have accessibility one stage prior to the corresponding change in gene expression (false discovery rate [FDR] <0.05; Figure 3B, F; Figure 3—figure supplement 1; Supplementary files 3, 9, and 12). Notably, nearly one-third of DACRs (1146) change accessibility between precompetent and competent larvae, with 71.4% becoming less accessible at competence. This extensive chromatin remodelling contrasts sharply with the relatively modest number of transcriptional changes between these stages (Figure 1G), and suggests that many chromatin changes precede settlement and may contribute to establishing a permissive genomic state for the rapid transcriptional activation required at the onset of metamorphosis.

Consistent with this interpretation, 62% of all genes that are differentially expressed in 1 hps postlarvae (3032) have proximal chromatin regions already accessible in competent larvae (Supplementary files 3 and 9). However, there is no significant difference in the rate of differential expression between genes associated with newly opened OCRs and those associated with other OCRs (9.84% vs 11.47%; *χ*²(1)=1.76, p = 0.184). In addition, during the first hour of metamorphosis, 65% of the 1555 DACRs become more accessible, a proportion closely matching the fraction of genes that are transcriptionally repressed at this stage (63%; Figures 1G and 3F). This correspondence is consistent with a model in which pioneer-like and other highly expressed TFs regulate both the acquisition of competence and the initiation of metamorphosis by interacting with their cognate binding motifs in OCRs proximal to DEGs.

### *CLOCK*, *Jun*, and *Fos* OCRs are enriched in binding motifs of highly expressed TFs

Given that many of the highly expressed TFs are differentially expressed either at competence or at 1 hps and that more than 90% of TFBMs enriched in larval and postlarval OCRs are potential binding sites of these highly expressed TFs (Figures 2A, B, 3D, E; Supplementary files 6 and 10), we chose to analyse the OCRs associated with three TF genes that are differentially expressed and appear central to the acquisition of larval competence or the initiation of metamorphosis: *CLOCK*, *Jun*, and *Fos*. bHLH and bZIP TFBMs are abundant in OCRs associated with genes that remain constantly expressed across these stages (Supplementary file 10), reinforcing the view that CLOCK and AP-1 occupy central positions in the regulatory networks governing both competence acquisition and environmentally induced cellular reprogramming at the onset of metamorphosis.

We analysed the *CLOCK* gene because it (1) is the most significantly and transiently upregulated TF gene in competent larvae at sunset, consistent with a light-dependent role in establishing competence, and (2) is a pioneer-like TF that can be affected by environmental light in other animals (Figure 2A; Supplementary file 6; Doi et al., 2006; Iwafuchi-Doi and Zaret, 2016; Jindrich et al., 2017; Menet et al., 2014). bHLH-PAS and bHLH (E-box) family motifs consistent with CLOCK:BMAL/CLK-like activity are present in OCRs associated with 165 and 1121 DEGs at competence and 1 hps, respectively (Supplementary file 13). This widespread distribution of bHLH-PAS/E-box family motifs is consistent with pioneer-like regulatory activity associated with CLOCK-related programmes that may contribute to establishing a permissive genomic state for rapid transcriptional responses to the algal inductive cue.

**Figure 4.**
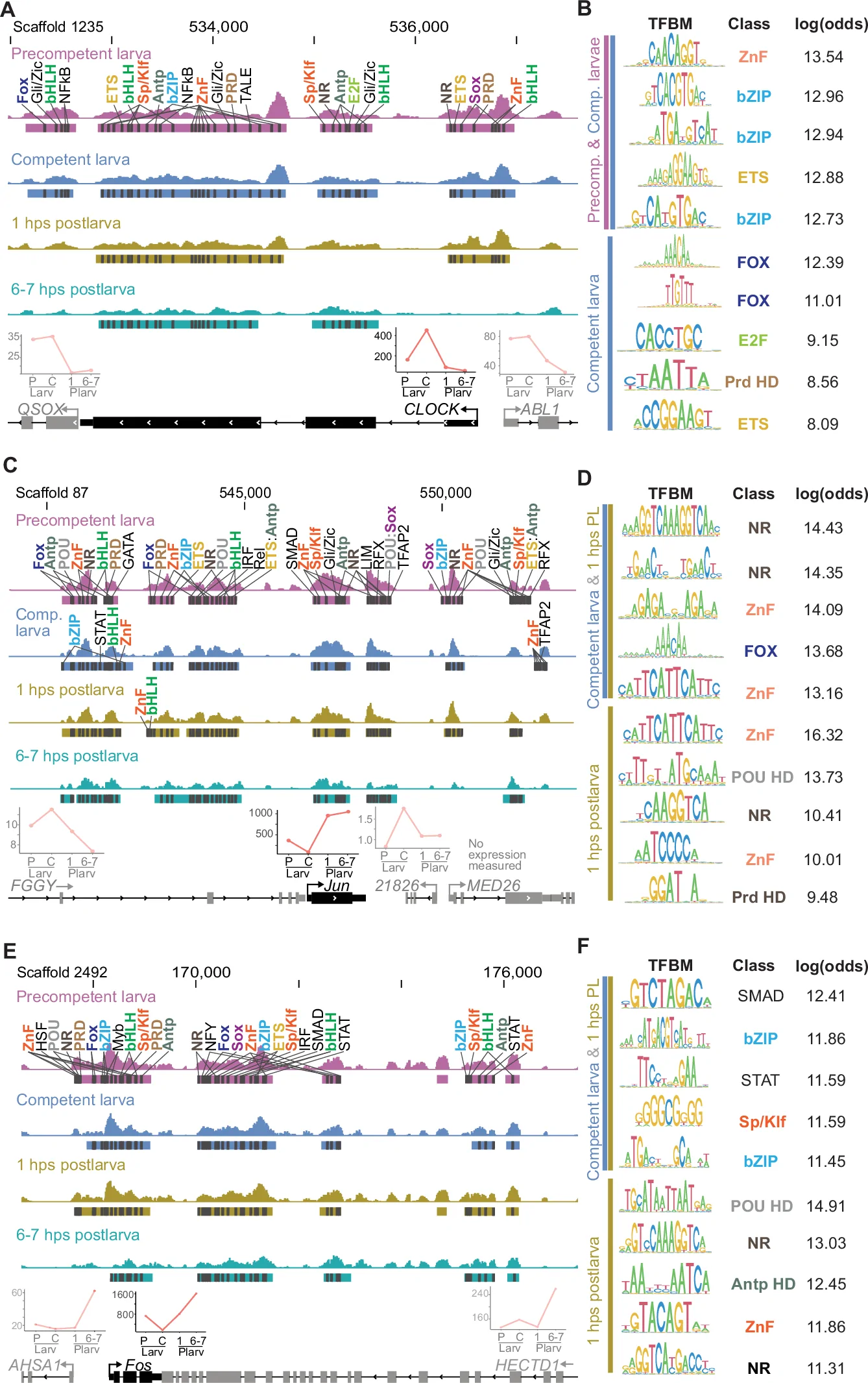
TFBMs enriched in dynamic open chromatin regions (OCRs) and in *CLOCK*, *Jun*, and *Fos*. (**A**) TFBM families mapped to *CLOCK* proximal OCRs (coloured bars under ATAC-seq peaks) in larval and postlarval stages (Supplementary file 15). The locations of the putative TFBMs are demarcated by a black bar and annotated at the stage at which the OCR first appears. TFBMs colour-coded in bold correspond to highly expressed transcription factor (TF) families. Log (odds) scores are positively correlated with motif match confidence. Protein-coding gene models at the bottom. Arrows, TSSs; chevrons, direction of transcription; exons, thick lines; UTRs, intermediate lines; introns, thin lines. The expression profiles are above the three genes (DESeq2 normalised counts). (**B**) The TFBM families in *CLOCK* OCRs with the highest matches with defined motifs (higher log-odds ratio being more likely a functional binding site). (**C**) TFBM families mapped to *Jun* proximal OCRs (Supplementary file 16). See description in (**A**) for details. (**D**) The TFBM families in *Jun* OCRs with the highest matches. See description in (**B**) for details. (**E, F**) TFBM families mapped to *Fos* proximal OCRs (Supplementary file 17). See descriptions in (**A, B**) for details.

In a genomic context, the *CLOCK* gene is flanked by protein-coding genes that do not show significant upregulation at competence (Figure 4A, B). Multiple OCRs flanking and within the *CLOCK* locus are enriched for TFBMs of highly expressed TF families, including Sp/Klf and other zinc-finger, bHLH, and Antp (Supplementary file 15). Notably, the two closest flanking OCRs become less accessible following settlement, coincident with *CLOCK* downregulation, suggesting that they function as activating *cis*-regulatory elements. CLOCK, together with many TFs predicted to bind these regulatory regions, is enriched in larval epithelial cell types that express light-detecting cryptochromes (Rivera et al., 2012; Supplementary file 7), consistent with a role in responding to diminishing light at sunset and being associated with the acquisition of competence.

AP-1 components Jun and Fos, together with other potential bZIP partners (CEBP, CREB, PAR, MAF, and NFE2) (Jindrich and Degnan, 2016; Reinke et al., 2013; Rodríguez-Martínez et al., 2017), are significantly and transiently upregulated during the first hour of metamorphosis. At 1 and 6–7 hps, 470 and 340 DEGs, respectively, have AP-1 or related bZIP TFBMs within associated OCRs, including numerous other TFs and conserved developmental regulators (Supplementary file 14). Jun and Fos play a conserved role in early immediate transcriptional responses to diverse endogenous and exogenous signals, often via MAPK signalling pathway (Bejjani et al., 2019; Eferl and Wagner, 2003; Shaulian and Karin, 2002). MAPK signalling is necessary for *A. queenslandica* settlement and metamorphosis (Ueda et al., 2016). We therefore focussed on the OCRs in the vicinity of these two genes.

*Jun* and *Fos* are both flanked by genes with distinct expression dynamics (Figure 4C–F). OCRs associated with both loci are enriched for binding motifs of TFs that are highly expressed during competence and early metamorphosis (Supplementary files 16 and 17). These include multiple bHLH and bZIP motifs, consistent with coordinated activity of bHLH and bZIP family TFs during the induction of *Jun* and *Fos* at the onset of metamorphosis, and with Jun, Fos and other bZIP factors contributing to their downregulation at competence and again at 6–7 hps. With the exception of one flanking OCR at each locus that becomes inaccessible at 6–7 hps, chromatin accessibility in the vicinity of *Jun* and *Fos* remains largely stable during larval development and early postlarval stages, suggesting that transcriptional dynamics at these loci are primarily driven by changes in TF availability rather than wholesale chromatin remodelling.

### CLOCK and other competence-associated TFs are repressed when larvae are prevented from experiencing sunset

**Figure 5.**
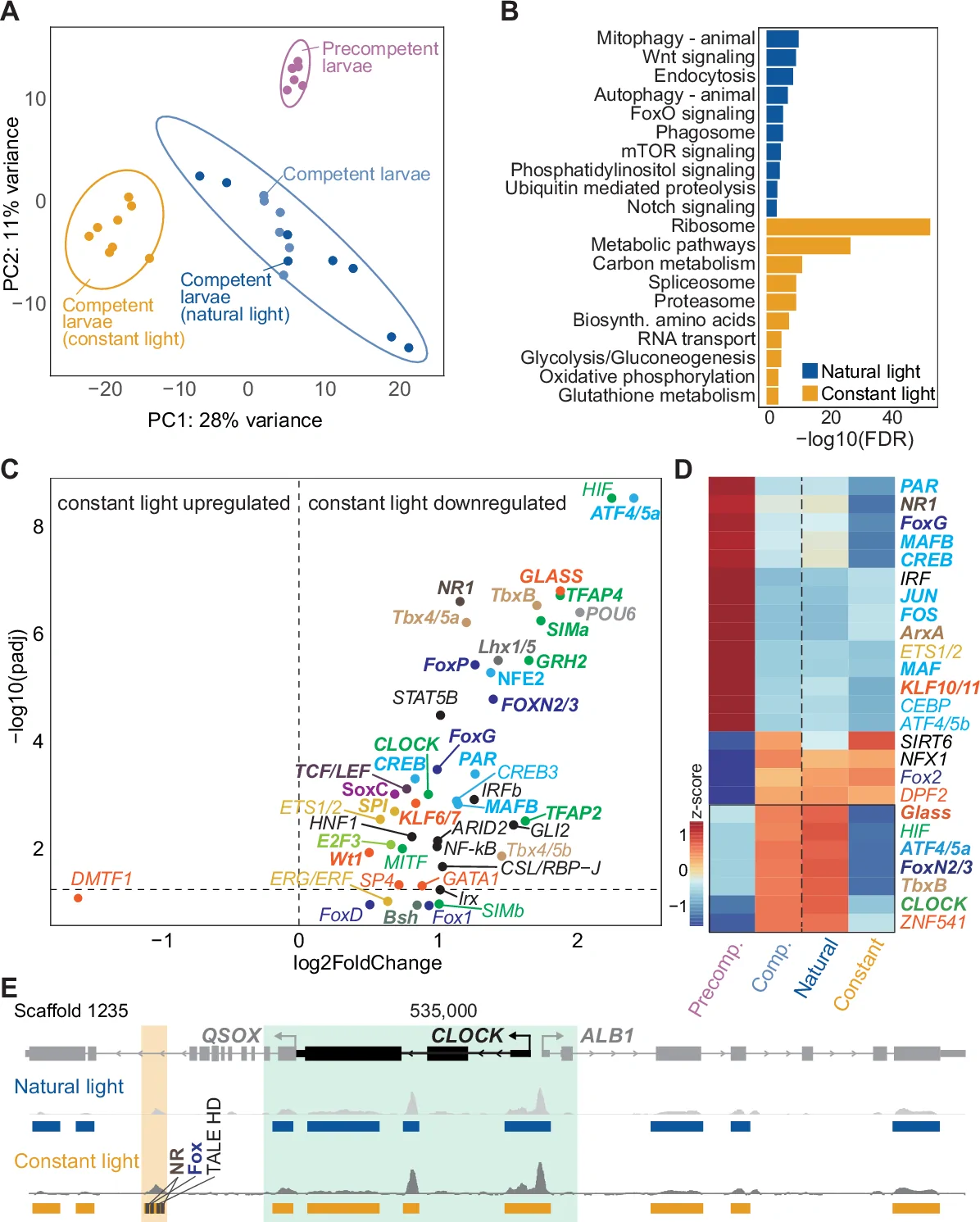
The effect of constant light on larval gene expression and chromatin state. (**A**) PCA showing the relationship of normal transcriptomes of precompetent and competent larvae (Figure 1D), and larvae exposed to natural and constant light. (**B**) Ten top KEGG categories in larvae exposed to natural and constant light (Supplementary file 18). (**C**) Volcano plot of transcription factors (TFs) up- and downregulated in larvae exposed to constant light; bolded TFs are expressed in the top 5% of all genes. (**D**) Scaled heatmap of 25 TF genes that are normally differentially expressed between precompetent and competent larvae, compared to their expression in natural and constant light. TFs that are normally markedly upregulated in competent larvae but repressed by constant light are boxed (bottom). The TFs that are within the top 5% are in bold. (**E**) Proximal *CLOCK* open chromatin regions (OCRs) do not change in light-exposed larvae (green box; see Figure 4A), but a downstream chromatin region in a QSOX intron becomes more accessible in larvae exposed to constant light (tan box). TFBMs present in this OCR are shown as per Figure 4.

To test the role of diminishing light at sunset in regulating gene expression during the acquisition of larval competence, we exposed larvae to constant light beyond sunset, a treatment that inhibits settlement and metamorphosis (Say and Degnan, 2020). Relative to larvae that experienced natural diminishing light, constant light-exposed larvae differentially up- and downregulated 779 and 879 protein-coding genes, respectively (DESeq2; p-adj <0.1), activating metabolic pathways that are not normally expressed in swimming larvae (Figure 5A, B; Supplementary file 18). This transcriptional profile is distinct from both precompetent and competent larval states. Importantly, we have previously shown that larvae exposed to constant light beyond sunset swim normally, and will rapidly settle and initiate metamorphosis as normal once they are transferred into darkness (Say and Degnan, 2020), suggesting that the constant light phenotype is not due to abnormal physiology or stress; no stress pathways are enriched in larvae exposed to constant light (Supplementary file 18).

Strikingly, TF expression was broadly repressed under constant light: 45 TFs were significantly downregulated and only a single TF was upregulated (Figure 5C). Consistent with this widespread decrease in TF expression, 88% of the OCRs present in larvae under natural light conditions became inaccessible in constant light-exposed larvae (Supplementary file 18). Together, these results indicate that exposing larvae to constant light beyond sunset is associated with a novel transcriptional and chromatin state that is distinct from normal developmental trajectories, and extinguishes their capacity to respond to the algal cue, thereby preventing settlement and metamorphosis.

Several TFs that are normally upregulated and highly expressed in competent larvae, including CLOCK, HIF, TboxB, FoxN2/3, ATF4/5, and Glass (with CLOCK, TboxB, FoxN2/3, ATF4/5, and Glass ranking in the top 5% of expressed genes, and HIF in the top quartile), were all significantly downregulated in constant-light-exposed larvae (Figure 5D). In addition to HIF, the bHLH-PAS TF SIMa, a potential partner of CLOCK, was also highly expressed in competent larvae and significantly repressed under constant light. Consistent with CLOCK and its potential partners having pioneer-like regulatory properties related to metamorphosis, a large proportion (24%) of the OCRs that close in constant light-exposed larvae have bHLH-PAS and E-box family motifs.

**Figure 6.**
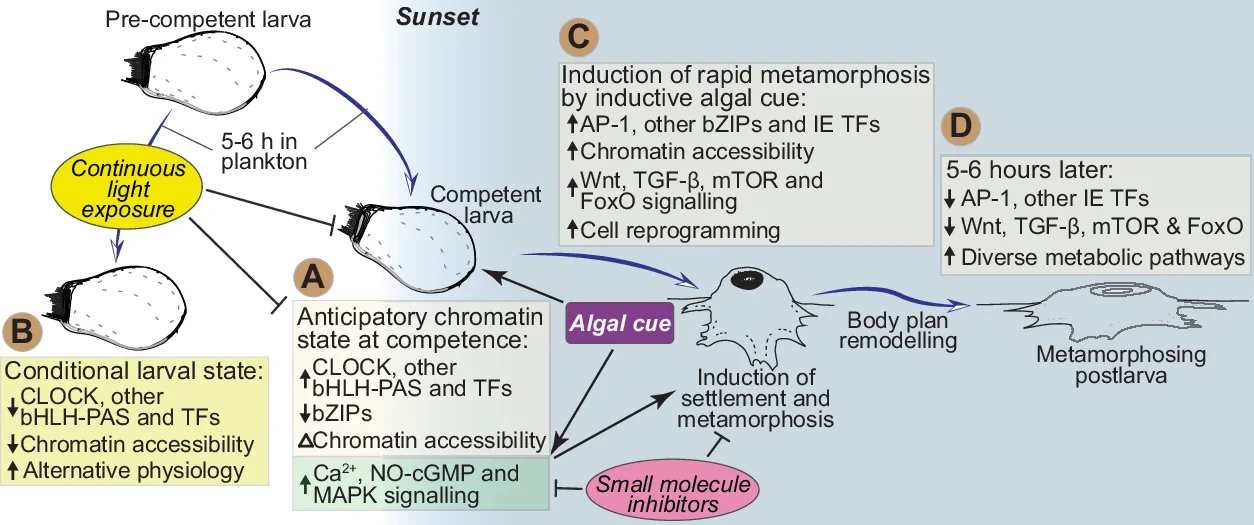
Environmental cues, genomic regulatory processes, and signalling events underlying larval competence and early metamorphosis in *A. queenslandica*. This model combines results from this and previous studies (Nakanishi et al., 2015; Say and Degnan, 2020; Ueda et al., 2016). (**A**) Pre-competent larvae emerge from adults early in the afternoon, then swim in the water column for several hours before becoming competent to settle and initiate metamorphosis immediately after sunset (Degnan and Degnan, 2010; Say and Degnan, 2020). With the acquisition of competence is (1) the upregulation of genes encoding CLOCK and other bHLH–PAS transcription factors (TFs), and other TFs, and the downregulation of AP-1 and other bZIPs; and (2) a change in chromatin accessibility in the vicinity of genes that are differentially expressed at competence and in the first hour of metamorphosis. (**B**) Larvae prevented from experiencing sunset do not settle in the presence of inductive algae nor initiate metamorphosis (Say and Degnan, 2020). Chromatin accessibility in light-exposed larvae decreases markedly and larvae move into an alternative physiological state and remain in the plankton. This includes the repression of over 40 TF genes, including *CLOCK* and *HIF*. (**C**) A cue on the surface of the alga *A. fragilissima* induces an internal signalling cascade that includes activation of calcium (Ca^2+^), nitric oxide (NO-cGMP) and MAPK pathways; larvae exposed to pharmacological agents that inhibit these pathways do not settle and metamorphose (Nakanishi et al., 2015; Song et al., 2021; Ueda et al., 2016). Newly settled postlarvae undergo large changes in chromatin accessibility and gene expression within the first hour of settling, repressing larval genes and activating multiple developmental signalling pathways and over 60 TFs, including AP-1 and other potential partner bZIP TFs, and other conserved Immediate-early (IE) TFs. During this first hour there are rapid and extensive morphogenetic changes with multiple cell types being reprogrammed (Blard et al., 2026). (**D**) 5–6 hr later, most developmental TF and signalling genes are downregulated, and diverse metabolic genes are activated, indicative of an overall shift in postlarval developmental and physiological state.

Examination of chromatin accessibility across the CLOCK locus and within 100 kb of its genomic position revealed that the proximal OCRs normally associated with *CLOCK* at competence (Figure 4A) were unchanged under constant light, whereas six distal regulatory regions exhibited altered accessibility, with five becoming less accessible (Supplementary file 18). Notably, the sole region that increased in accessibility in constant-light larvae lies within an intron of an adjacent gene approximately 2.5 kb downstream of *CLOCK* (Figure 5E). These rapid, light-dependent changes in chromatin state support the existence of a conditional gene regulatory program that is normally suppressed at sunset. When activated by aberrant light conditions this program is associated with the downregulation of *CLOCK* and other competence-associated TF genes, disrupting chromatin priming, and preventing the transcriptional and chromatin changes required for larval competence, settlement and metamorphosis (Figure 6).

## Discussion

Metamorphosis is one of the most dramatic and consequential transitions in an animal’s life, transforming planktonic larvae specialised for dispersal and environmental sensing into benthic juveniles/adults adapted for growth, survival and reproduction. In *A. queenslandica*, this environmentally induced transition is remarkably rapid, unfolding within hours of settlement. We show that this swift morphological transition is underpinned by an unexpectedly rapid and large transcriptional response in the first hour of metamorphosis, with the expression level of nearly 4,900 genes changing significantly. Such speed implies that genomic regulatory processes must be initiated before the inductive cue is encountered. Here, by integrating time-resolved transcriptomic and chromatin accessibility profiling across larval competence, settlement, and early postlarval development, we show that larvae undergo extensive regulatory reorganisation while still swimming in the plankton, prior to metamorphic induction. In particular, diminishing light at sunset is associated with widespread changes in chromatin accessibility that precede large-scale transcriptional activation at settlement (Figure 6). These findings indicate that the larval genome enters an anticipatory state that is permissive for rapid, coordinated transcriptional reprogramming, rather than responding de novo to the inductive environmental cue (Nakanishi et al., 2015; Say and Degnan, 2020; Ueda et al., 2016).

A defining feature of *A. queenslandica* metamorphosis is the immediate and substantial activation of a large suite of deeply conserved developmental TFs. To our knowledge, such a diverse suite of TFs reaching expression levels within the top 5% of all genes has not been observed in other animal developmental transitions. Their exceptionally high expression at competence and at the onset of metamorphosis may confer regulatory robustness, ensuring sustained occupancy of *cis*-regulatory elements and enabling rapid, coordinated shifts in gene expression. Among these, the AP-1 components Fos and Jun, together with their potential bZIP heterodimeric partners (Reinke et al., 2013; Rodríguez-Martínez et al., 2017), are rapidly and strongly induced following settlement. AP-1 is frequently activated downstream of MAPK and is a conserved effector of environmentally responsive signalling pathways that regulate cell differentiation, proliferation and death in diverse animals (Bejjani et al., 2019; Eferl and Wagner, 2003; Shaulian and Karin, 2002). The strong enrichment of bZIP motifs within OCRs associated with genes that change expression immediately after settlement supports a central role for AP-1 in coordinating the transcriptional reprogramming underlying metamorphosis. Consistent with this interpretation, inhibition of MEK/ERK signalling blocks settlement and metamorphosis in *A. queenslandica* and other marine invertebrate larvae (Cavalcanti et al., 2020; Heyland and Moroz, 2006; Shikuma et al., 2016; Taylor and Heyland, 2018; Ueda et al., 2016). Thus, although the timing of competence acquisition and the identity of inductive settlement cues are largely species-specific, the initiation of metamorphosis itself may rely on a conserved regulatory logic involving MAPK signalling and AP-1-mediated transcriptional activation.

The induction of metamorphosis in *A. queenslandica* is conditional on the prior acquisition of competence, which develops in larvae after a period in the plankton and exposure to diminishing light at sunset (Degnan and Degnan, 2010; Say and Degnan, 2020). We show here that competence acquisition is accompanied by extensive remodelling of chromatin accessibility and changes in gene expression. These changes not only appear to permit settlement in response to the algal cue, but also establish a permissive chromatin landscape that enables the rapid and large-scale transcriptional response observed during the first hour of metamorphosis (Figure 6). Larvae prevented from experiencing sunset fail to settle and metamorphose, exhibit markedly reduced expression of CLOCK and multiple other highly expressed developmental TFs, and display widespread loss of chromatin accessibility. Together, these findings support a model in which CLOCK and associated pioneer-like TFs mediate anticipatory chromatin priming that is required for the subsequent induction of AP-1 and other developmental regulators at settlement.

Diminishing light intensity has been implicated in regulating larval settlement in a range of marine species (Thorson, 1964), raising the possibility that circadian entrainment of competence via CLOCK represents a widespread and ancestral feature of the pelagobenthic life cycle. Notably, *A. queenslandica* larvae maintained in constant light do not simply remain in a precompetent state but instead transition into an alternative transcriptional and chromatin configuration that is distinct from both precompetent and competent larvae. This observation suggests the existence of sensitive, rapidly deployable and conditional gene regulatory programs that are normally suppressed under predictable sunset conditions. Activation of such alternative programs may allow larvae to delay settlement and extend dispersal when environmental conditions are unfavourable, thereby modulating connectivity and colonisation potential.

Together, our results support a model in which environmentally predictable planetary cues, such as the daily light–dark cycle, are integrated during the planktonic larval phase to establish a permissive genomic state that enables rapid induction of metamorphosis upon encountering a suitable, often species-specific, settlement cue. In *A. queenslandica*, this state is characterised by (1) extensive chromatin remodelling prior to settlement, (2) transient but exceptionally high expression of conserved developmental TFs at both competence and the initiation of metamorphosis, and (3) enrichment of binding motifs of these TF families in regulatory regions associated with genes regulated during early metamorphosis. Functional dissection of individual regulators is currently unfeasible in *A. queenslandica*. Nevertheless, the temporal ordering of chromatin accessibility changes, TF expression, and environmental induction suggests light-entrained transcriptional activity – potentially involving CLOCK-associated and AP-1/bZIP family factors – contributes to chromatin priming and rapid transcriptional reprogramming. We propose that such anticipatory chromatin regulation may represent a general solution to the problem of speed and robustness in metamorphosis, enabling biphasic animal life cycles to integrate predictable planetary signals with species-specific ecological cues across evolutionary timescales. In the absence of equivalent chromatin-resolved datasets from parahoxozoan lineages (e.g. echinoderms, hemichordates, annelids, molluscs, bryozoans, or cnidarians), the extent to which the mechanisms identified here are conserved remains an open question.

## Methods

### Larval and postlarval collection and cell isolation

Adult *A. queenslandica* were collected from the intertidal reef flat of Shark Bay, Heron Island Reef, Southern Great Barrier Reef (23° 27′S, 151° 55′E) (Great Barrier Reef Marine Park Authority Permit G22/46272.1) and were immediately transferred to aquaria at Heron Island Research Station, where they were maintained in flow-through, unfiltered ambient seawater drawn from the adjacent reef flat (Leys et al., 2008). Naturally emerging larvae from 12 randomly collected adults were randomly collected in the early to mid-afternoon, and maintained in ambient conditions as previously described (Say and Degnan, 2020). Randomly selected larvae were transferred at sunset (5–6 hours post-emergence; hpe) to a 6-well dish with 10 ml of 0.22 µm filtered seawater (FSW) (10 per well), and then exposed 30 min later to three small branches of the coralline algae *A. fragilissima* as previously described (Say and Degnan, 2020). These dishes were maintained under ambient water temperature and light conditions (Say and Degnan, 2020). For collection of postlarval stages, dishes were checked every 30 min to determine the time of settlement, defined by the larva remaining attached to the alga when the dish was given a gentle shake.

Six randomly sampled larvae and postlarvae were collected from each of the following stages for cell dissociation, and CEL-seq2 and ATAC-seq analyses: precompetent larvae (0–1 hpe); competent larvae (5–6 hpe); 1 hps postlarvae; 3–4 hps postlarvae; 6–7 hps postlarvae; and 11–12 hps postlarvae (Figure 1D). All individuals were treated individually (i.e. not pooled). Six randomly sampled larvae and postlarvae per stage were collected for CEL-seq2; three of these were also used for ATAC-seq. All postlarvae that had settled on *A. fragilissima* were removed rapidly from the alga with a sharp tungsten needle and transferred into FSW on ice. Precompetent and competent larvae were treated the same but did not need to be removed from the alga. All individual larvae and postlarvae were washed three times for 2 min in ice-cold FSW and then centrifuged through a 25-µm metal mesh fitted into a 1.5-ml Eppendorf at 500 × *g* for 10 min at 4°C. Pelleted fragments and cells were resuspended and washed with ice-cold 0.22 µm filtered calcium-magnesium-free artificial seawater (CMFSW; 449 mM NaCl, 33 mM Na_2_SO_4_, 2.15 mM NaHCO_3_, 9 mM KCl, 10 mM Tris-HCl pH 10, and 2.5 mM EGTA) for 5 min and recentrifuged at 500 × *g* for 10 min at 4°C. The cell pellet was resuspended in 20 µl ice-cold CMFSW. This procedure took less than 25 min at 4°C, thus minimising changes in gene expression or chromatin state associated with these perturbations.

For four developmental stages – precompetent and competent larvae, and 1 and 6–7 hps postlarvae – we separately assayed both gene expression and chromatin accessibility in the same individuals by splitting their resuspended cells into two pools. For each individual of these four stages, 5 and 15 µl of the suspended cells were used for CEL-seq2 and ATAC-seq library construction, respectively. For CEL-seq2, 5 µl of the cell suspension was immediately mixed with 50 µl ice-cold TRIzol and stored in –80°C. For ATAC-seq, 15 µl of the cell suspension was immediately mixed with 50 µl ice-cold ATAC-seq cell lysis buffer and processed as described below. The other two stages – 3–4 and 11–12 hps postlarvae – were assayed only for gene expression, so that the entire 20 µl of the cell suspension was immediately mixed with 200 µl ice-cold TRIzol, resulting in the same dilution as above, and stored in –80°C.

### Imaging early metamorphosis

Images of *A. queenslandica* postlarvae were taken with a Nikon SMZ25 microscope using the program Nikon Elements and 6X zoom every 3 min, starting from ~10 min after the initiation of settlement on *A. fragilissima* through to ~2 hps. Each time point consisted of a z-stack with 14 images at an interval of 25 µm, which were then stacked into a single image using the focus stacking program Zerene Stacker with the P Max algorithm (Brecko et al., 2014). These were cropped, brightened and denoised, then unsharp masked using Adobe Photoshop. To generate the timelapse movie, the frames were compiled into an avi using ImageJ and converted to mp4 (or mkv) using AnyVideoConverter (https://www.any-video-converter.com/en8/for_video_free/).

### CEL-seq2 library construction, sequencing, and analysis

RNA isolation and quality-assessment, and CEL-seq2 library construction all were performed as previously described (Hashimshony et al., 2016; Hashimshony et al., 2012; Sogabe et al., 2019; Yuan et al., 2025). Libraries were sequenced on two lanes of Illumina HiSeq-PE 150 platform and raw sequencing data were processed using a publicly available pipeline (https://github.com/yanailab/celseq2, YanaiLab, 2018; Hashimshony et al., 2016) with a built-in suite of python-based scripts for demultiplexing, mapping to the genome, and counting reads. Raw reads were mapped to *A. queenslandica* Aqu3.1 genome (https://www.ncbi.nlm.nih.gov/bioproject/?term=PRJNA668660) (Xiang et al., 2022) using Bowtie2 (Langmead and Salzberg, 2012), and assessed for coverage and quality as previously described (Say and Degnan, 2020; Sogabe et al., 2019; Supplementary file 1). Count tables for the two sequencing lanes were merged using a custom R script (https://github.com/hfyuanuq/thesis.scripts, copy archived at Yuan, 2026b) before the differential expression analysis. CEL-seq2 raw counts from the two larval ages in two different light regimes were obtained from https://www.ncbi.nlm.nih.gov/bioproject/?term=PRJNA534516 (Say and Degnan, 2020) and larval single-cell RNA-seq raw counts were obtained from https://www.ncbi.nlm.nih.gov/bioproject/?term=PRJNA435744 (Sebé-Pedrós et al., 2018).

### Analysis of DEGs

Only genes with at least 4 read counts in at least four samples were considered expressed; those not meeting these criteria were filtered out from all subsequent analysis. PCA of all expressed genes was performed on variance-stabilising transformation (VST) transformed counts and visualised using the function ‘plotPCA’ to examine sample-to-sample distances and identify potential outliers with 95% confidence ellipses. Differential gene expression was conducted in R (R Development Core Team, 2021) using the Bioconductor package DESeq2 (Love et al., 2014). Pairwise comparisons were conducted between each of the six developmental stages to produce a DEG list for each pair of stages (including 1 hps vs 6–7 hps, to match the successive ATAC-seq stages), with an FDR threshold of <0.1 (Supplementary file 3). This permissive FDR was chosen to detect changes in transcript abundance within 1 hr (i.e. between competent larvae and newly settled 1 hps postlarvae), given known eukaryotic rates of RNA transcription and degradation (Sun et al., 2012). Venn diagrams were generated using the R package ggVennDiagram (Gao et al., 2021). Heatmaps were produced in R using packages pheatmap (Kolde, 2025) and RColorBrewer (Neuwirth, 2022) to visualise expression profiles with rlog normalised counts for CEL-seq2 datasets. Single-cell RNA-seq reads were normalised with the function CPM (*cpm(data, log = TRUE*)) using the Bioconductor package edgeR (version 3.14.0) implemented in R (Robinson et al., 2010). Alluvial (Sankey) diagrams were generated using SankeyMATIC (vBeta https://sankeymatic.com/build/).

### Co-expression analysis

WGCNA (version 1.61) (Langfelder and Horvath, 2008) was performed on the VST-transformed developmental dataset using R (version 4.0). We used a signed Nowick-type topological overlap metric with soft threshold of *β* = 3. Co-expression modules were defined using dynamic tree cut (height 0.15; minimum module size 30). Module eigengenes were correlated with time points. To retain the distinct expression patterns of each module, a merging distance threshold of 0.1 was applied.

### ATAC-seq library preparation, sequencing, and analysis

ATAC-seq libraries were prepared as previously described (Cornejo-Páramo et al., 2022; Yuan et al., 2025). Briefly, we added ATAC-seq cell lysis buffer (10 mM Tris-HCl, pH 7.4, 10 mM NaCl, 3 mM MgCl_2_, 0.1% vol/vol IGEPAL CA-630) to the 15 µl of dissociated cells (15–20,000 cells) and centrifuged at 500 × *g* for 10 min at 4°C. The pellet was resuspended in 50 µl transposition reaction and incubated at 37°C for 30 min. The resultant DNAs were amplified, purified, and quality assessed as previously described (Cornejo-Páramo et al., 2022). Libraries were sequenced on a 2 × 101 bp NovaSeq SP flow cell platform. Raw reads were trimmed and low-quality reads removed using FastQC and Trim-Galore (parameters: -q 30 --phred33 --stringency 4 --length 20 -e 0.1) (https://github.com/FelixKrueger/TrimGalore, Krueger et al., 2026). Reads were mapped to the *A. queenslandica* Aqu3.1 genome using Bowtie2 (parameters: -X 1000) (Langmead and Salzberg, 2012). Paired reads were identified using SAMtools (parameter: -f 2) (Li et al., 2009; Danecek et al., 2021) and duplicated reads were identified using Sambamba and removed (Tarasov et al., 2015). MACS2 was used to call peaks (parameters: -g 1.2e8 --nomodel --shift -100 --extsize 200) (Zhang et al., 2008). This yielded over 46 million reads that corresponded to over 20,000 peaks from each library (Supplementary file 8). ATAC-seq library quality was determined by calculating fraction of reads in peaks (FRiP) scores using the ENCODE ATAC-seq pipeline (https://www.encodeproject.org/atac-seq/); an FRiP score over 0.2 was deemed acceptable (Supplementary file 8). Peaks from the three replicate ATAC-seq libraries for each stage were pooled and overlapping peaks were intersected using bedtools (Quinlan and Hall, 2010), also in the ENCODE ATAC-seq pipeline (https://www.encodeproject.org/atac-seq/).

### Analysis of OCRs

ATAC-seq peaks were annotated using the Bioconductor package ChIPseeker (Yu et al., 2015), with the promoter region set as ±500 bp (Supplementary file 9). The Bioconductor package DiffBind (V2.16.0) was used for differential binding analysis of peaks (Stark and Brown, 2011). Significantly different peaks (FDR <0.05) between the four developmental stages were identified by pairwise comparisons on successive stages using DESeq2 (Love et al., 2014). Consistent peaks and overlapping peaks were generated in DiffBind using the function dba.peakset (parameters: consensus = DBA_FACTOR, minOverlap = 0.33) (Stark and Brown, 2011). Library correlation analysis was performed using ChromVAR (Schep et al., 2017) to determine peaks across all 12 libraries. Genes associated with these peaks were compared to the list of genes associated with ChIP-seq peaks (Gaiti et al., 2017).

### Motif identification, annotation, clustering, and accessibility analysis

Homer (Heinz et al., 2010) was used to identify TFBMs with parameters of -size 500 -len 6, 8, 10, 12 -h. All identified motifs were combined into a single motif list, and we excluded all the potential *A. queenslandica* specific, sequence bias motifs, and non-significant identified motifs (p-value >1e^–10^). Motifs were matched to the database (https://jaspar2022.genereg.net/downloads/) that we customised by removing fungi- and plant-specific motifs (available at https://github.com/hfyuanuq/atac-pub, copy archived at Yuan, 2026a) using MEME-TomTom (version 5.5.8) with default threshold 0.5 (Gupta et al., 2007). After motif matching, all putative TFBMs were assigned with JASPAR motif identifiers. Then motifs were clustered into TF classes and families based on JASPAR annotations using the Bioconductor package TFBSTools (Tan and Lenhard, 2016).

TF-binding sites analysis was performed using the GimmeMotifs scan tool (Bruse and Heeringen, 2018; van Heeringen and Veenstra, 2011). OCR sequences of expressed genes and DEGs were scanned against the JASPAR non-redundant database. Motif scan significance was determined using log-odds scores calculated relative to the *A. queenslandica* genomic background model. Putative motif occurrences were filtered using an FDR threshold of 0.01. Following the scan, all putative TFBMs were clustered into TF class and family as previously described. Chromatin accessibility and peak profiles were visualised using the R package Gviz (Hahne and Ivanek, 2016) and the Integrative Genomics Viewer (IGV) software (Thorvaldsdóttir et al., 2013).

### Enrichment analyses

KEGG enrichments, which are threshold-dependent, were performed using the online tool STRING (https://string-db.org/) with default settings. To more fully capture functions that are enriched among highly regulated genes, rather than only among highly expressed genes, we also conducted a GO_MWU (Mann–Whitney *U* test for Gene Ontology term enrichment) analysis, based only on fold-change (Voolstra et al., 2011). Available at https://github.com/z0on/GO_MWU, Matz, 2022.

### Light perturbation experiment

From 12 adults collected and maintained in flow-through seawater aquaria at Heron Island Research Station, as described above,>50 naturally released larvae were collected within 30 min of emerging, and split equally among two 500 ml glass beakers of sea water. One beaker was exposed to natural light and the other to constant light, both at ambient temperature, as described previously (Say and Degnan, 2020). At sunset (5–6 hpe), eight larvae from each beaker were randomly collected. Their cells were dissociated and CEL-seq2 and ATAC-seq libraries were constructed, sequenced and analysed as described above (Yuan et al., 2025).

CEL-seq2 and ATAC-seq sequencing were performed using the Illumina NovaSeq X Series sequencer (PE150, NovaSeq Control Software v1.2.2.48004 and Real Time Analysis v4.6.7). Precompetent and competent larval and constant and natural light CEL-seq2 datasets were combined and integrated, with competent larval and natural light samples being combined into a single group. Batch effects were corrected using ComBat-Seq (Zhang et al., 2020). Differential OCRs and associated TFBMs were identified, characterised, and visualised as described above. For each motif, the average deviation difference between constant and natural conditions was computed to determine the direction and magnitude of activity change.

### Use of AI tools

Claude was used to improve our text for overall readability and grammatical corrections. We verified every edit for scientific accuracy.

### Materials and correspondence

Materials and correspondence should be addressed to S.M.D. or B.M.D.

## Data Availability

All data generated or analysed during this study have been deposited in NCBI BioProjects database. CEL-seq2 and ATAC-seq data for normal light condition are available at accessions PRJNA1162246 and PRJNA1161795, respectively, and for constant light condition at accessions PRJNA1394668 and PRJNA1394227, respectively. Source data files have been provided for all figures as supporting files. The following datasets were generated: YuanH DegnanBM DegnanSM 2024Transcriptomes from larval and early postlarval stages of the demosponge Amphimedon queenslandicaNCBI BioProjectPRJNA1162246 YuanH DegnanBM DegnanSM 2024ATAC-seq data from larval and early postlarval stages of the demosponge Amphimedon queenslandicaNCBI BioProjectPRJNA1161795 YuanH DegnanBM DegnanSM 2025Transcriptomes from the demosponge Amphimedon queenslandica swimming larvae that exposed under different light conditionsNCBI BioProjectPRJNA1394668 YuanH DegnanBM DegnanSM 2025ATAC-seq data from the demosponge Amphimedon queenslandica swimming larvae exposed to constant light and natural light conditionsNCBI BioProjectPRJNA1394227 The following previously published dataset was used: XiangX DegnanBM DegnanSM 2020Amphimedon queenslandica and its bacterial symbiontsNCBI GenBankGCA_016292275.1

## Additional files

Supplementary file 1. Information on CEL-seq2 libraries.

Supplementary file 2. Normalised and average CEL-seq2 counts for all expressed genes using DESeq2.

Supplementary file 3. Genes that are differentially expressed during larval development and early metamorphosis.

Supplementary file 4. KEGG enrichments for genes that are upregulated at each larval and early postlarval stage and GO-MWU enrichments of differentially expressed genes between successive stages.

Supplementary file 5. KEGG enrichments for genes in each WGCNA modules.

Supplementary file 6. TF genes that are expressed and differentially expressed during larval development and early metamorphosis.

Supplementary file 7. Raw and normalised MARS-seq counts (Sebé-Pedrós et al., 2018) of differentially expressed TF genes in larval cell types.

Supplementary file 8. Information on ATAC-seq libraries: sequencing reads, mapping ratios, peaks, and fractions of reads in called peak regions.

Supplementary file 9. Genomic location and TFBMs enrichment in OCRs present during larval development and early metamorphosis. Larval enhancers and promoters inferred by ChIP-seq (Gaiti et al., 2017) overlapped with OCRs identified in precompetent larvae in the present study.

Supplementary file 10. OCRs and their TFBMs associated with expressed genes at all four stages.

Supplementary file 11. OCRs and their TFBMs associated with WGCNA modules.

Supplementary file 12. OCRs and their TFBMs associated with DEGs between precompetent and competent and competent and 1 hps.

Supplementary file 13. OCRs and their PAS related (bHLH) TFBMs associated with differentially expressed genes during larval development and early metamorphosis.

Supplementary file 14. OCRs and their Fos/Jun-related (bZIP) TFBMs associated with differentially expressed genes during larval development and early metamorphosis.

Supplementary file 15. OCRs and TFBMs found in and around *CLOCK* during larval development and early metamorphosis.

Supplementary file 16. OCRs and TFBMs found in and around *Jun* during larval development and early metamorphosis.

Supplementary file 17. OCRs and TFBMs found in and around *Fos* during larval development and early metamorphosis.

Supplementary file 18. Comparison of gene expression, chromatin state and accessible TFBMs in larvae exposed to natural and constant light.

MDAR checklist
