# Supplementary material for: Light-entrained chromatin priming poises rapid metamorphosis in a marine sponge
Source: eLife. 2026 Oct 27;15:RP111060. doi: 10.7554/eLife.111060 (PMC13713027; doi:10.7554/eLife.111060)
Supplement: Peer review history

# Editor report: eLife Assessment

**DOI:** 10.7554/eLife.111060.3.sa0

**Authors:** Gáspár Jékely

**Affiliations:** Heidelberg University Germany

**Keywords:** Compelling, Fundamental

In this study, Yuan and colleagues carry out transcriptomic and epigenomic experiments to study open chromatin regions and transcripts that change upon larval settlement in the sponge Amphimedon. The authors present **compelling** evidence to show that sponge larvae prepare for receiving an environmental cue (sunset) by extensively modifying their chromatin accessibility. The study represents a **fundamental** advance in understanding the fine genetic control of larval settlement and has significance beyond the immediate field of sponge larval biology.

# Referee report: Reviewer #1 (Public review)

**DOI:** 10.7554/eLife.111060.3.sa1

Summary:

Yuan and colleagues present a thorough study of gene activation before and during metamorphosis in sponge larvae, combining in depth analyses of staged transcriptomes and chromatin accessibility profiling (ATACseq). Amongst several very interesting findings, the study reveals that the acquisition of settlement competence, which arises in response to decreasing light at sunset, is characterized by changes in chromatin accessibility that anticipate strong transcriptional shifts occurring as metamorphosis starts. Another notable finding is a set transcription factors amongst the genes strongly up-regulated at the onset of metamorphosis. In addition, larvae exposed to constant light, a condition that stalls metamorphosis, were found to activate metabolic pathways that are not normally expressed in swimming larva, Together the findings provide a rare level of understanding into how environmental conditions can promote deployment of alternative developmental programs in planktonic larvae.

Strengths:

This is a comprehensive and rigorous study of a phenomenon of wide interest. It will inspire researchers working on other species to look for similar, environmentally-driven "anticipatory" epigenetic mechanisms. It also provides a wealth of detailed information on genes, notably transcription factors, that are candidates for involvement in regulating specific metamorphosis transitions - and beyond. The data presented here are thus undoubtedly a rich and valuable resource.

Weaknesses:

It is not always straightforward to connect the conclusion statements in the text to the figures, or to grasp the aims and logic of the workflows.

# Referee report: Reviewer #2 (Public review)

**DOI:** 10.7554/eLife.111060.3.sa2

Summary:

It is demonstrated that sponge larvae prepare for receiving the environmental cue (sunset) by extensively modifying their chromatin accessibility in the absence of large gene expression changes - "anticipatory" chromatin remodeling. This program can be offset by modifying the cue (making light constant), leading to a novel molecular state. These are the most important novel findings. Then, around metamorphosis there is dramatic regulation of transcription factors and their targets. While this result is hardly surprising, it is nice to see it demonstrated thoroughly in a phylogenetically fundamental animal study system.

Strengths:

This is a top-notch study of a key life cycle transition in an organism of great phylogenetic importance, involving concurrent gene expression and chromatic accessibility profiling (to the best of my knowledge, this has never been done in non-bilaterians and likely anywhere outside Vertebrata). The result is highly non-trivial. There is also an additional experiment modifying the key environmental cue (constant light), adding additional insight.

Weaknesses:

The paper presents a great amount of material, which somewhat obscures results related to the major novel finding ("anticipatory" chromatin remodeling). Among them is the fact that there is no significant statistical association between regions opened during remodeling and genes subsequently regulated during metamorphosis. This lack of direct association suggests that there may be more to the story than just priming the genome for metamorphosis.

# Referee report: Reviewer #3 (Public review)

**DOI:** 10.7554/eLife.111060.3.sa3

Summary:

In their manuscript, Huifang Yan and colleagues perform RNA-seq (CEL-seq) and ATAC-seq experiments to profile the transcriptome and chromatin accessibility of sponge larvae across larval competence, settlement and early postlarval development. Amphimedon, the sponge species that they use, is amenable to lab experiments and can therefore be a convenient model for experimenting with this otherwise difficult to assay ecological parameters and cues. They had previously observed that light conditions (diminished light) at sunset are Sucritical for larvae to enter a pre-settlement stage and prime them for settlement and metamorphosis. In this paper they report that these conditions induce a gain of accessibility in many genes, including transcription factors, and that altering these conditions by providing continuous light at sunset affects this reprogramming event.

Strengths:

The above is a very interesting observation, one that the authors speculate that could have a broader significance and be a theme in many more larvae. I agree with the authors that this is an important finding and I think that the paper will be interesting for the broad readership of eLife. If this is the case, the authors open up a new theme of chromatin regulation, extensively studied in mammalian contexts, but severely understudied in pretty much every other context.

Weaknesses:

I think however that their paper often reports the data in a difficult to follow way, and that other sorts of analyses would have made the results more accessible for the broad readership.

# Author comment: Author response

**DOI:** 10.7554/eLife.111060.3.sa4

**Authors:** Huifang Yuan, Oceane Board, Zac Pujic, Bernard M Degnan, Sandie M Degnan

**Affiliations:** University of Queensland; University of Queensland; The University of Queensland St Lucia Australia; The University of Queensland Brisbane Australia; The University of Queensland Brisbane Australia

The following is the authors’ response to the original reviews.

**Public Reviews:**

**Reviewer #1 (Public review):**

Summary:

Yuan and colleagues present a thorough study of gene activation before and during metamorphosis in sponge larvae, combining in-depth analyses of staged transcriptomes and chromatin accessibility profiling (ATACseq). Amongst several very interesting findings, the study reveals that the acquisition of settlement competence, which arises in response to decreasing light at sunset, is characterized by changes in chromatin accessibility that anticipate strong transcriptional shifts occurring as metamorphosis starts. Another notable finding is a set of transcription factors amongst the genes strongly up-regulated at the onset of metamorphosis. In addition, larvae exposed to constant light, a condition that stalls metamorphosis, were found to activate metabolic pathways that are not normally expressed in swimming larvae. Together, the findings provide a rare level of understanding into how environmental conditions can promote deployment of alternative developmental programs in planktonic larvae.

Strengths:

This is a very comprehensive, well-documented and rigorous study of a phenomenon of wide interest. It will inspire researchers working on other species to look for similar, environmentally-driven "anticipatory" epigenetic mechanisms. It also provides a wealth of detailed information on genes, notably transcription factors, that are candidates for involvement in regulating specific metamorphosis transitions - and beyond. The data presented here are thus undoubtedly a rich and valuable resource.

We thank reviewer #1 for finding our study on sponge metamorphosis interesting and compelling, and that it is likely to inform future studies on gene regulation and activity in environmentally regulated developmental processes and metamorphosis.

Weaknesses:

I see no significant weaknesses; however, the documentation of the data is very compressed, with all the findings contained in 4 multi-panel figures with succinct legends. It is not always straightforward to connect the conclusion statements in the text to the figures. Although the relevant data is available in supplementary files, I would appreciate more help in navigating the data to assess the support for key conclusions, if possible, illustrating each text conclusion explicitly in the main figures.

Thank you sincerely for these suggestions on how to better present the results. We agree that the figures and associated legends are succinct. To rectify this in the hope of improving clarity and accessibility, we have (i) created two new figures by splitting our original four figures into six, and (ii) expanded figure legends to provide more explanatory details. We also made minor additions to the main Results text to more fully explain some results (see also reviewer #3’s comments).

Specifically, we:

(1) Removed panel K (heat map of TF expression) from original Fig. 1 and created a new figure (new Fig. 2) that focuses solely on TF expression and emphasises the extraordinarily high expression of many TFs. We also moved into the new Fig. 2 a panel from the original SFig. 1 that documents the larval cell types that express these most highly expressed abundant TF transcripts. This new figure should provide the reader with a clearer perspective on high TF expression in the larval competence and the initiation of metamorphosis.

(2) Removed panel F from the original Fig. 4 (now Fig. 5) to create a new, expanded Figure 6 that presents a stand-alone summary of the main findings of this work; that is, environmental regulation of competence and early metamorphosis. This allowed us to (i) incorporate the constant light experiment into the summary figure, and (ii) provide a more detail explanation in the legend.

**Reviewer #2 (Public review):**

Summary:

It is demonstrated that sponge larvae prepare for receiving the environmental cue (sunset) by extensively modifying their chromatin accessibility in the vicinity of genes that are going to be regulated during metamorphosis, in the absence of large gene expression changes. This program can be offset by modifying the cue (making light constant), leading to a novel molecular state.

Strengths:

This is a top-notch study of a key lifecycle transition in an organism of great phylogenetic importance, involving concurrent gene expression and chromatic accessibility profiling (to the best of my knowledge, this has never been done in non-bilaterians and likely anywhere outside Vertebrata). The result is highly non-trivial. There is also an additional experiment modifying the key environmental cue (constant light), adding additional insight.

We thank reviewer #2 for their efforts and for appreciating the approaches we employed to understand environmental induction of sponge metamorphosis. In addition to the phylogenetic importance of sponges, their pelagobenthic life cycle is likely shared with disparate bilaterians (but not with vertebrates and other chordates, whose metamorphoses are probably derived).

Weaknesses:

I have only a couple of suggestions.

(1) Not all new pre-emptively opened OCR regions are associated with genes that are going to be regulated during metamorphosis. Is their association with such genes statistically significant? (Fisher's exact test?)

Thank you for raising this helpful point. In following your suggestion to statistically test this, we determined that a Fisher’s Exact Test was not appropriate because that test is generally used only for small samples or tables with expected counts below 5; in our data, all four expected cell frequencies are well above 5 (minimum = 228.6) and N = 25,149. Thus we instead tested for an association between chromatin accessibility and differential gene expression using the more appropriate Pearson's chi-squared test. We found no significant difference in DEG rate between genes associated with newly opened OCRs and those associated with other OCRs (9.84% vs 11.47%; χ^2^(1) = 1.76, p = 0.18), and have added these details into the Results (lines 344-46) as follows: “Consistent with this interpretation, 62% of all genes that are differentially expressed in 1 hps postlarvae (3032) have proximal chromatin regions already accessible in competent larvae (Supplementary Tables 3 and 9), although statistically we find no significant difference in DEG rate between genes associated with newly opened OCRs and those associated with other OCRs (9.84% vs 11.47%; χ^2^(1) = 1.76, p = 0.184).”

(2) Re: extended discussion on possible reasons for activation of specific transcription factor families. I feel it is not terribly useful since it is hardly more than guesswork. The authors should consider condensing this part to better emphasize the major (and most unexpected) large-scale regulation patterns.

We agree with this appraisal and have modified the beginning of Discussion to highlight the large-scale and rapid changes of overall gene expression. This emphasises the regulatory processes – TF expression and chromatin state changes – that must be in place to allow such transcriptional changes to occur. We feel this addition enhances the focus on TF activation and regulation at competence and early metamorphosis, especially given the scale and level of change, with most of TFs being expressed at very high levels (i.e. top 5% of all gene expressed). As outlined above, we created a new figure focussed on highly expressed TFs (new Fig. 2) to hopefully further highlight this phenomenon. It would be of great interest to know if this is conserved amongst animals with a pelagobenthic life cycle and rapid metamorphosis.

(3) Re: enrichment analysis based on significant genes (Figure 1H): Even though it is a common practice, there is nuance: as we all know very well, many genes pass a significance threshold not because they are highly differentially regulated (i.e., show large fold-change), but because they are more abundantly expressed overall and so the statistical power for them is greater. A good example is ribosomes - before we realized what was happening, they would show up as enriched in almost every experiment of ours, which was not very useful since their fold-change was quite trivial. I see the authors have ribosome enrichment too, and I suspect there are a few more functional groups that made it because they tend to express highly on average. Ideally, we want to see what is enriched among highly regulated genes, not among abundantly expressed genes. Because of this we moved to compute enrichment based only on fold-change, using the GO_MWU package (https://github.com/z0on/GO_MWU). I suggest authors give it a shot, to see if the enrichment results become more interpretable. GO_MWU is also very powerful to analyze enrichment in WGCNA modules, in case the authors want to try that.

Thank you for this interesting insight and advice. We applied the GO_MWU package to our gene expression dataset. Overall, these new results corroborated the original KEGG enrichment analysis, largely identifying GO biological processes, cellular components and molecular functions consistent with the previously identified KEGG molecular and cellular processes operating at larval competence and 1 hps. These include genomic regulatory processes underlying transcriptional changes and morphogenetic processes that occur in the first hour of metamorphosis, and which are also highlighted in a recent BioRxiv paper (https://doi.org/10.64898/2026.04.23.719999) from our group.

We have added (i) results from the GO MVU analyses to Supp. Fig. 1 and Supp. Table 4, (ii) the following statement to Fig. 1 legend: “GO-MWU analysis of upregulated genes reveal stage-specific enrichments largely consistent with the KEGG analysis (Supplementary Fig. 1 and Supplementary Table 4).”, and (iii) a brief description of this approach into the Methods.

**Reviewer #3 (Public review):**

Summary:

In their manuscript, Huifang Yan and colleagues perform RNA-seq (CEL-seq) and ATAC-seq experiments to profile the transcriptome and chromatin accessibility of sponge larvae across larval competence, settlement and early postlarval development. Amphimedon, the sponge species that they use, is amenable to lab experiments and can therefore be a convenient model for experimenting with this otherwise difficult to assay ecological parameters and cues. They had previously observed that light conditions (diminished light) at sunset are critical for larvae to enter a pre-settlement stage and prime them for settlement and metamorphosis. In this paper, they report that these conditions induce a gain of accessibility in many genes, including transcription factors, and that altering these conditions by providing continuous light at sunset affects this reprogramming event.

Strengths:

The above is a very interesting observation, one that the authors speculate could have a broader significance and be a theme in many more larvae. I agree with the authors that this is an important finding, and I think that the paper will be interesting for a broad readership. If this is the case, the authors open up a new theme of chromatin regulation, extensively studied in mammalian contexts, but severely understudied in pretty much every other context.

We thank reviewer #3 for their positive assessment of our findings, pointing out the novelty of this research and its broad relevance.

Weaknesses:

I think, however, that their paper often reports the data in a difficult-to-follow way, and that other sorts of analyses would have made the results more accessible for a broad readership. Here, I present some suggestions that the authors might want to take into account to improve their results.

Reviewer #1 also commented on how the results were difficult to follow. Based on your and their comments, we have reworked parts of this section, and the figures and legends. Details of these changes are listed above and can be viewed in the new version with track changes on.

We note that no further specific suggestions were visible to us in your review.
